# Process Phase Estimation and Deviation Detection for Manual Soldering Based on Motion Analysis

**DOI:** 10.3390/biomimetics11090618

**Published:** 2026-09-01

**Authors:** Kyohei Wakabayashi, Tetsuya Oda

**Affiliations:** 1 Graduate School of Engineering, Oita University, 700 Dannoharu, Oita 870-1192, Japan; v24f2005@oita-u.ac.jp; 2 DX Human Resource Development Foundation Program, Department of Science and Technology, Faculty of Science and Technology, Oita University, 700 Dannoharu, Oita 870-1192, Japan

**Keywords:** task phase estimation, deviation detection, soldering, three-dimensional pose estimation

## Abstract

Manual soldering requires phase-dependent coordination of posture, hand movement, tool position, and visual attention. We propose a depth-camera framework that estimates the work phase and detects deviations using three-dimensional upper-body and hand features, task-related object positions, and gaze-related approximation features derived from facial orientation and head posture. The system estimates three predefined phases: preparation, active soldering, and cleanup. Windows with insufficient phase confidence are assigned to Uncertain Phase and routed to review rather than treated as deviation labels. In the evaluation, normal trials showed stable process sequences, whereas trials with scripted simulated unsafe-like movements produced local increases in the deviation score and review-required intervals associated with reduced phase-estimation reliability. These findings suggest that the framework may support retrospective safety-related assessment and the identification of process-inconsistent operations in seated manual soldering under controlled laboratory conditions. The bio-inspired contribution is a functional abstraction of phase-dependent perceptual-motor coordination into context-dependent engineering reference patterns and an uncertainty-aware review mechanism.

## 1. Introduction

Manual soldering involves rapid transitions among approach, heating, material supply, and withdrawal at small solder joints. Posture control, hand movement, tool positioning, and attention directed toward the workpiece are closely intertwined during these operations. Therefore, a support system for this task must be capable not only of assessing the quality of the finished product but also of tracking the sequence of steps through which the task is performed over time. In practice, what is required on the shop floor is not merely an evaluation of the final result after task completion but also an understanding of the postures and tool manipulations performed at each stage, from preparation through the main task to cleanup. This perspective is important not only for stabilizing work quality but also for training support, skill transfer, and safety support during the task.

Furthermore, manual soldering involves multiple thermal and ergonomic hazards. Since hot soldering irons are handled near the face and hands, there is a risk of burns and unintended contact. In addition, seated soldering often involves a forward-leaning posture, neck flexion, static holding of the upper limbs, and repetitive fine hand movement. A relationship between these factors and musculoskeletal strain in electronics work has been reported [1,2,3]. Therefore, safety-related work analysis requires not only detecting atypical postures or movements but also evaluating whether those observations are appropriate in light of the current phase. For example, although bringing a soldering iron close to a circuit board is necessary during soldering, the same movement may be inappropriate during preparation or cleanup.

For soldering in high-reliability applications, JAXA JERG 0 039D(E) [4] systematically defines process requirements and quality standards. However, although this standard specifies appropriate procedures and finished states, it does not address working posture, tool position, spatial relationships with the workpiece, or line of sight during the process. To provide safety and skill support on-site, the workflow assumed by the standards must be mapped to measurement data obtained from cameras so that the process context and observed deviations can be interpreted within a single system.

### Related Studies and Research Gap

Technologies for estimating human pose from camera images have evolved from two-dimensional pose estimation to three-dimensional movement analysis. Cao et al.’s Part Affinity Fields provided a foundation for real-time pose estimation under natural lighting conditions [5], and subsequently, the MediaPipe family has been widely used as a practical estimation pipeline capable of handling the body, hands, and face simultaneously [6,7,8,9]. In the field of ergonomics, posture information has been used to evaluate physical strain and work-related disorders. Rapid Upper Limb Assessment (RULA) is a representative method for rapidly evaluating upper limb load and posture-related risk [10], and Alipoor et al. demonstrated that motion capture and digital human modeling are effective for designing manual assembly workstations [1]. Yang et al. reported a correlation between musculoskeletal disorders and unnatural posture and repetitive movement among electronics manufacturing workers [2], while Sillanpää et al. demonstrated the importance of workstation design for tasks involving high visual workload [3]. These studies indicate that posture information is effective for evaluating work safety and physical strain. In recent years, efforts have been made to directly integrate posture estimation into observation-based ergonomic evaluation and to automatically calculate scores such as RULA from images. For example, methods for estimating RULA evaluation levels from RGB images have been proposed, demonstrating the potential for continuous monitoring while reducing observer subjectivity and the burden of prolonged observation [11,12]. Furthermore, configurations that incorporate risk assessment through posture classification have also been reported [13]. On the other hand, it has been pointed out that the validity of assessment in actual manufacturing environments can vary depending on environmental factors [14], and the ability to estimate posture does not necessarily imply the ability to perform reliable on-site risk assessment. Furthermore, systems have been proposed to select evaluation methods, such as RULA, Rapid Entire Body Assessment (REBA), or Ovako Working Posture Analysis System (OWAS), based on joint angles and task type, and calculate them in real time [15]; however, these approaches primarily focus on mapping posture to workload. Additionally, in task analysis research based on posture estimation, the primary focus is often on recognizing specific postures or movement patterns. However, in precision manual tasks such as soldering, even when the same forward-leaning posture or hand proximity is observed, the meaning of the movement changes depending on the process phase. Therefore, task analysis for soldering requires a system that can simultaneously address not only posture detection itself but also the process phase in which that posture occurs.

Understanding manual tasks requires not only body movement but also awareness of tools, materials, and the state of the workpiece. You Only Look Once (YOLO) [16] and Mask Region-based Convolutional Neural Network (Mask R-CNN) [17] are standard methods for object detection and segmentation and are widely used in industry. In the manufacturing sector, Oyekan et al. tracked the progress of manual assembly sequences using depth cameras [18], while Chen et al. recognized repetitive assembly movement by combining object detection and pose estimation [19]. Furthermore, Kastner et al. integrated object detection and pose estimation to support augmented reality [20], and Coronado et al. estimated fine-grained assembly actions based on multi-view observations [21]. These studies demonstrate that task understanding requires both body movement and object relationships. Recent studies have increasingly moved from isolated posture or action recognition toward multimodal and temporally structured analysis of manual assembly. Wang and Yan integrated skeletal and object information for assembly-action recognition and progress prediction [22], while Chen et al. combined RGB observations, hand-skeleton information, and temporal features for fine-grained continuous assembly-activity classification [23]. Multimodal datasets for manufacturing assembly and methods that incorporate tool recognition as contextual information have also been reported [24,25]. Recent work has further extended vision-based worker analysis toward realistic manufacturing environments. Agostinelli et al. evaluated computer-vision-based ergonomic assessment across different real manufacturing workstations [14], and Tomelleri et al. integrated multiple sensing modalities for digital ergonomic assessment in a human-centric manufacturing framework [26]. More recently, markerless vision has been directly compared with inertial and marker-based motion capture for ergonomic assessment [27], while Papoutsakis et al. presented a vision-based framework for three-dimensional worker behavior, ergonomic analysis, and fine-grained activity recognition in industrial assembly lines [28]. In particular, fingertip location, tool position, and the visibility of the target object are crucial clues for determining task progress. On the other hand, much of the existing research primarily focuses on recognizing process progress or providing task assistance, and the identification of safety-relevant deviations is often secondary. Furthermore, even when safety is addressed, many approaches independently detect high-risk postures or proximity to hazards, and few configurations evaluate these observations on the premise that their significance varies with the process phase. In soldering tasks, the soldering iron, solder wire, and circuit board are in close proximity, and the worker’s hands and face are also located nearby. Therefore, distances between objects and visibility ratios are relevant to both process understanding and safety assessment. Consequently, this study does not treat object recognition as merely supplementary information but instead positions it as an observable used for both process estimation and deviation detection.

JAXA JERG 0 039D(E) [4] specifies requirements covering multiple stages of the soldering process, including preparation, heat application, solder application, cleaning, and inspection. The most important implication of these standards for this study is that soldering is not a collection of individual actions but a sequence of operations in which meaning varies depending on the specific stage of the process. For example, while bringing the soldering iron close to the circuit board is necessary during the actual soldering phase, a similar approach during preparation or cleanup may be interpreted as an inappropriate operation. In other words, the acceptability of a given action is not determined by the action alone; its meaning changes depending on which process stage is currently being performed. The European standard ECSS Q ST 70 61C organizes assembly processes, including soldering, as conditional work procedures for the assembly and verification of high-reliability electronic circuits [29]. Furthermore, IPC J-STD-001J provides requirements for general industry, including soldering materials, process conditions, and acceptability, indicating that soldering is a task that presupposes process control [30]. On the other hand, ergonomic studies have reported that posture adopted during seated work varies not only with body shape but also with task characteristics such as manual work, visual work, and combined tasks [31]. Furthermore, it has been shown that unnatural and sustained posture in the neck and shoulder regions during assembly work can affect discomfort and work efficiency [32] and that ergonomic interventions in electronics and semiconductor assembly are effective in reducing musculoskeletal strain [33]. Therefore, the safety of soldering operations must be evaluated not only in terms of proximity to heat sources and tools but also in terms of which process stage and under what visual and manual conditions it is being performed and in which posture the action occurs. While standards provide process knowledge, this knowledge cannot be directly mapped to image recognition results or single-frame features. Image observations instead yield observables such as body posture, head orientation, hand position, distance between the tools and the board, and object visibility; these observables differ in form from the descriptions in the standards. Therefore, rather than directly applying standard knowledge to image features, it is necessary to first estimate the current process from the observed time series and then to interpret each observed quantity in light of that process.

Taken together, previous studies demonstrate that human posture, hand motion, task-related objects, and temporal information can be used for ergonomic assessment and manual-task understanding. However, these elements have generally been addressed as posture assessment, action recognition, or task-progress estimation, whereas their integration for phase-conditioned interpretation of deviations in precision manual work remains limited.

To date, the authors have studied the prediction of unsafe-like movements from three-dimensional posture, camera-placement optimization for posture changes during soldering, and attention-related posture detection using a depth camera [34,35,36]. These studies established a basis for task observation but did not model differences among process phases or interpret observed deviations according to the current phase.

Based on this research gap, this study develops a phase-conditioned RGB and depth (RGB-D) framework for seated manual soldering. The novelty of this study lies in the following three points: (1) a two-stage framework that first estimates the current soldering phase and then evaluates multimodal deviations using the reference pattern associated with that phase; (2) the integration of upper-body posture, hand motion, task-related object relationships, object visibility, and face-orientation-related observations; (3) the routing of low-confidence phase intervals to human review rather than assigning an automatic experimental-state label. The bio-inspired source of the proposed framework is the phase-dependent coordination of perception, movement, and tool handling observed in skilled human work. In skilled behavior, posture, hand motion, and tool-workpiece relationships are not interpreted as isolated signals; their functional meaning depends on the current task context. The proposed engineering realization transfers this organizing principle into multimodal sensing, task-phase inference, phase-conditioned reference interpretation, and explicit human review when the task context is uncertain. Thus, this study contributes to the advancement of biomimetics at the level of functional information-processing organization rather than by imitating biological morphology or neural mechanisms. The biological/behavioral source, its functional abstraction, and its engineering realization are therefore distinguished explicitly. Furthermore, the discrimination performance and interpretability of the proposed framework are evaluated by comparing normal trials with trials containing scripted simulated unsafe-like movements.

The remainder of this paper is organized as follows. Section 2 describes the RGB-D acquisition, feature extraction, process-phase estimation, and deviation-detection methods. Section 3 presents the experimental results for normal trials and trials involving simulated unsafe-like movements. Section 4 discusses the findings, biomimetic interpretation, sensing choices, application perspective, and limitations. Finally, Section 5 presents the conclusions.

## 2. Proposed System

**Figure 1:**
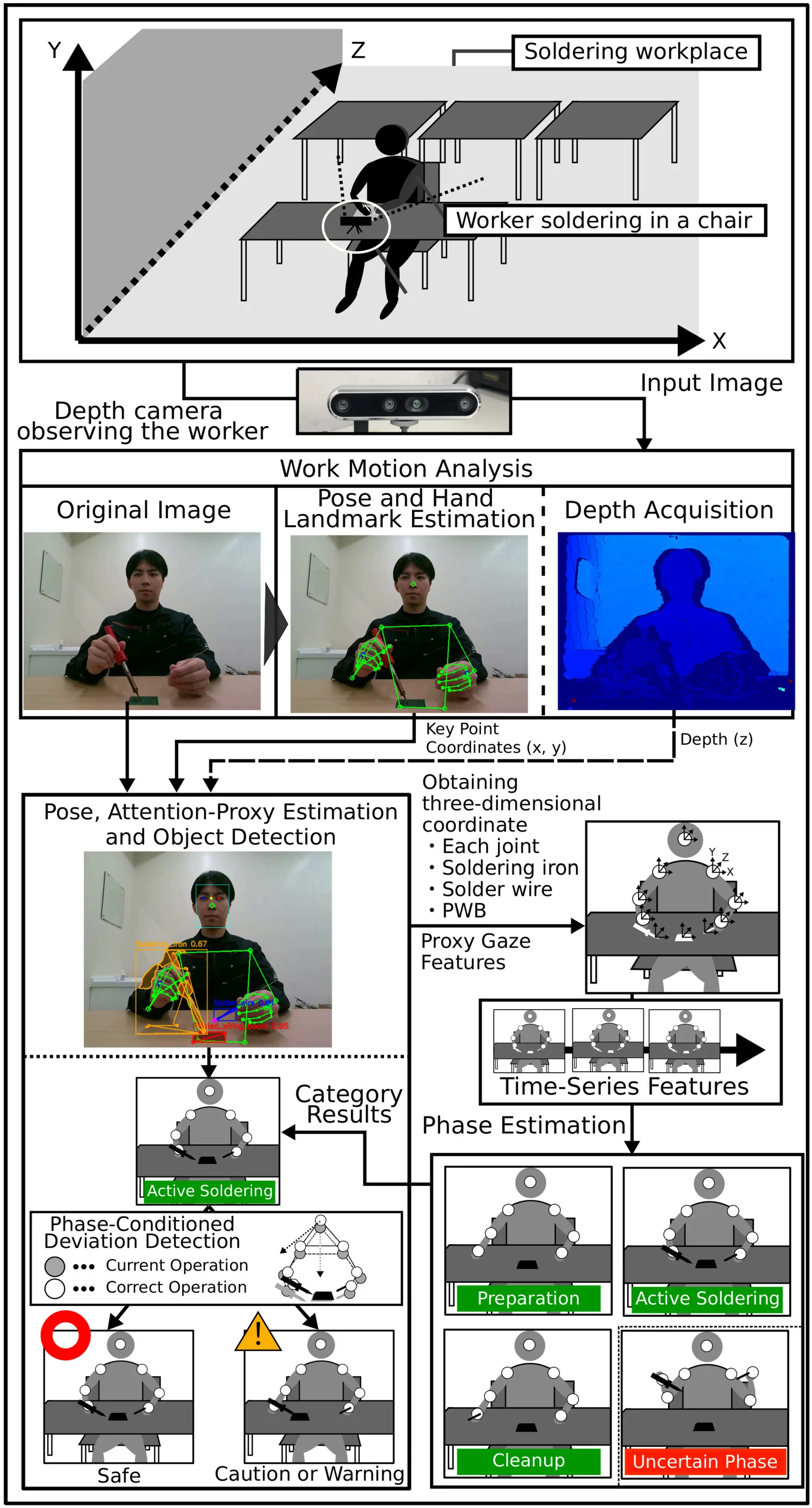
Overview of the proposed system.

**Figure 2:**
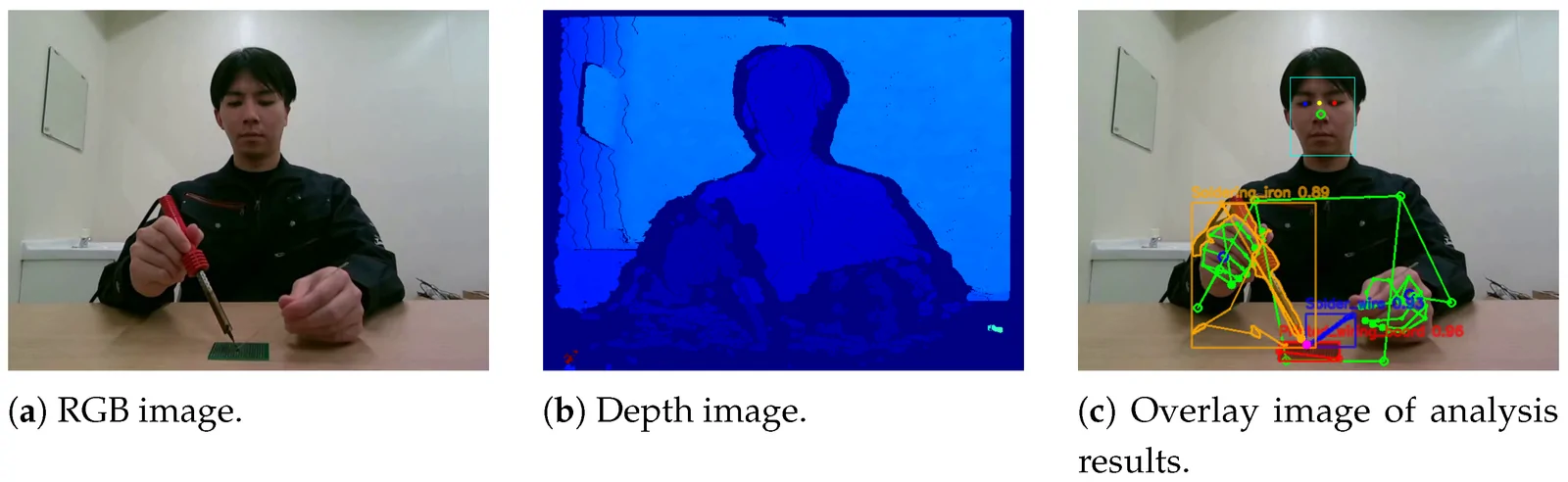
Example of acquired images and analysis results.

This section describes the proposed system. An overview of the proposed system is shown in Figure 1. The proposed system consists of five processing stages: (1) RGB-D acquisition; (2) body and hand feature extraction; (3) recognition of task-related objects and estimation of three-dimensional relationships; (4) process estimation; (5) deviation detection based on the estimated process. The input consists of synchronized RGB and depth images captured from the front during seated soldering tasks, and the output consists of the estimated task phase and deviation level at each time point. In this study, we adopted a hierarchical structure for process estimation and deviation detection. First, we estimated the current process based on temporal changes in body movement, tool placement, and facial orientation. Next, we evaluated deviations in posture, distance, and visibility in light of that process. We adopted this structure because, even when the geometric changes in landmarks observed during posture estimation are the same, such as when a soldering iron is brought close to a circuit board, the meaning of the movement can differ depending on the process. By prioritizing process estimation, we aimed to distinguish between approach movements required for the current task and inappropriate approaches that occur during preparation or cleanup. An Intel RealSense D455 camera was installed in front of the operator so that the face, upper body, both hands, and the workspace on the table were all included within the same field of view. RGB images were acquired at a resolution of 640×480 pixels in BGR8 format, whereas depth images were acquired at 640×480 pixels in Z16 format. Both streams were recorded at 30 fps, and the depth frames were aligned to the RGB coordinate system. The depth-measurement accuracy under the experimental configuration is summarized in Table 1. The intrinsic parameters were obtained from the active RealSense stream profiles, and the depth scale was approximately 0.001 m per depth unit. To examine depth accuracy under the experimental configuration, a planar target was recorded at known distances of 0.50, 0.60, and 0.70 m. Three independent trials were conducted at each distance. The mean measured distances were 0.4970, 0.5998, and 0.6937 m, respectively, corresponding to mean absolute errors of 3.00, 0.25, and 6.32 mm. The maximum relative bias was 0.90%, and the between-trial standard deviation was below 0.5 mm at all tested distances. RGB images were used for body landmark estimation, hand landmark estimation, face-related analysis, and object recognition, whereas depth images were used to project two-dimensional features into the three-dimensional workspace. Examples of the acquired RGB and depth images, together with the estimation results, are shown in Figure 2. Because the observations obtained from the acquired images contained short-term detection fluctuations at the frame level, the features were summarized for each time window and then used as input to the process estimator. Furthermore, for deviation detection, the data were re-aggregated at the one-second level so that the process and deviation sequences could be compared on the same time axis. By using three temporal scales (frame, time window, and second), the system preserved local movement differences while maintaining an overview of the entire process. In this study, the observations required for task understanding were categorized into four groups: body posture, hand movement, spatial relationships between tools and workpieces, and gaze-related approximation features. Gaze-related approximation features refer to a set of features that approximate gaze direction toward the work object based on facial orientation, head posture, and the geometric relationship with the area around the work surface. These observations were summarized for each time window and used for both process estimation and deviation detection.

### 2.1. Extraction of Three-Dimensional Posture and Hand Features

In seated manual soldering, upper limb posture and hand movement change in response to a sequence of operations, including approaching the circuit board, heating, supplying solder, withdrawing, and inspection. Therefore, by combining features that represent not only simple joint positions but also angles, velocities, finger opening and closing states, and bilateral hand coordination, the preparation, main task, and cleanup phases can be distinguished more clearly. For analysis of the operator’s three-dimensional body posture and hand features, body landmarks were estimated from RGB images, and the three-dimensional coordinates of each point were reconstructed on the basis of the corresponding depth values and camera intrinsic parameters. The main landmarks used were the nose, left and right shoulders, left and right elbows, left and right wrists, left and right hips, left and right knees, and left and right ankles. For local movement analysis, 21 landmark points on each hand were also used. The nose and both shoulders represent head and trunk orientation, whereas both elbows and both wrists represent upper-limb posture and task-related movement. The waist, knees, and ankles were used to assess trunk stability during seated posture. The following preprocessing steps were applied to the obtained three-dimensional joint sequences: (1) missing-value interpolation; (2) translation based on the waist midpoint; (3) scaling based on shoulder width and waist width; (4) normalization based on the tool-holding and solder-wire-holding hand roles. These procedures were introduced to reduce differences in body build and seating position relative to the camera among participants. Translation based on the waist midpoint was intended to focus on the relative movement of each body part rather than on translational movement of the whole body. Subsequently, the mean, standard deviation, minimum, maximum, median, interquartile range (IQR), intra-window variation, and mean absolute difference were calculated from the trajectories of the nose, shoulders, elbows, wrists, and hand centroids. The IQR was defined as the difference between the 75th and 25th percentiles and was used to represent variability within a time window while suppressing the influence of temporary outliers. In addition, elbow angle, wrist velocity, hand centroid velocity, hand opening, pinch width, inter-hand distance, wrist-to-nose distance, wrist height, wrist depth, and joint-loss rate were used as integrated features. These features characterize upper-limb coordination, postural stability, and manipulation variability.

### 2.2. Task-Related Object Recognition and Three-Dimensional Spatial Relationship Features

In addition to body movement, task-related object recognition was applied to RGB images to detect the printed wiring board (PWB), soldering iron, and solder wire. For each object, the centroid and representative points were extracted from the bounding box or mask, and the three-dimensional centroid and candidate endpoints were reconstructed by associating them with depth information. The PWB was treated as a reference object that defines the primary work area, whereas the soldering iron and solder wire were treated as manipulable objects with functional endpoints. The PWB was selected as the reference object because most of the work takes place near the board and the relative positions of the tools and the board are relevant to both process understanding and deviation assessment. As three-dimensional spatial relationship features, the system outputs the minimum distance between the face and the soldering iron, the minimum distance between the soldering iron and the PWB, the minimum distance between the solder wire and the PWB, the minimum distance between the wrist and the soldering iron, the minimum distance between the wrist and the solder wire, and the minimum distance between the tip of the soldering iron and the tip of the solder wire. These distances serve as indicators of facial safety, stability of tool handling, and coordination between tools. In addition, the system outputs the visibility ratio and median detection confidence for the PWB, soldering iron, and solder wire. Because a decrease in visibility ratio or detection confidence may reflect occlusion or inappropriate tool placement, these metrics were also used for deviation detection. From face-related analysis, the gaze estimation success rate, gaze approximation reliability, a flag indicating whether the gaze is directed near the PWB, and the head yaw, pitch, and roll angles were obtained. These were not direct gaze measurements but were treated as gaze-related approximation features representing the geometric alignment between face orientation and the work target. In soldering, fine manual operations must be performed while looking at the target surface. Therefore, face orientation and head tilt are important for both process estimation and deviation detection. The overall processing flow is shown in Algorithm 1. In object detection, the PWB, soldering iron, and solder wire were used as detection targets. The YOLO dataset comprised 1500 annotated images, with 500 annotated images for each of the three target classes. The images were obtained from five participants, with 300 images contributed by each participant. To avoid participant-level data leakage, the dataset was divided on a participant-wise basis: 900 images from three participants were used for training, 300 images from one participant were used for validation, and 300 images from the remaining participant were used for testing. No participant contributed images to more than one subset. YOLO11n-seg was used as the initial model and was trained for 100 epochs with an input image size of 640. The class-wise object results reported below correspond to segmentation-mask performance on the participant-disjoint test set. The training images and acquired data are not publicly available because they include the participants’ body postures and the experimental workspace.
**Algorithm 1** Process flow for task estimation and deviation detection based on the estimated task  1:Acquire synchronized RGB and depth images.  2:Extract body landmarks, hand landmarks, face-related features, and task-related objects from the RGB images.  3:Reconstruct three-dimensional joint features and three-dimensional spatial relationship features using the depth images.  4:Calculate posture features, spatial relationship features, object-recognition reliability features, and gaze-related approximation features from the frame-level data.  5:Estimate the posterior probabilities of the three predefined phases, Preparation, Active Soldering, and Cleanup, using the phase estimator.  6:Apply temporal smoothing to the phase probability sequence using the chronological workflow constraint Preparation → Active Soldering → Cleanup.  7:Aggregate the frame-level features and phase probabilities into 1.0 s intervals using median values.  8:Assign Uncertain Phase to an interval when the unavailable-phase ratio is high, the maximum smoothed phase probability is low, or the difference between the highest and second-highest phase probabilities is small.  9:Construct phase-conditioned reference ranges from normal trials by collecting one-second median features for each phase and defining the lower and upper bounds using the 5th and 95th percentiles.10:**for** each 1.0 s interval **do**11:   **if** the interval is assigned to Uncertain Phase **then**12:     Output the interval as a review-required interval.13:     Exclude the interval from automatic Safe/Caution/Warning deviation-level classification.14:   **else**15:     Calculate phase-specific deviation scores using the reference ranges of Preparation, Active Soldering, and Cleanup.16:     Calculate the final deviation score as the weighted sum of the phase-specific deviation scores using the smoothed phase probabilities.17:     Classify the interval into Safe, Caution, or Warning using thresholds tuned on the development data.18:   **end if**19:**end for**20:Visualize the time series of smoothed phase probabilities, review-required intervals, deviation scores, and deviation levels.

### 2.3. Estimation of Soldering Process Phases

In soldering process phase estimation, the temporal progression of the work is classified into three predefined phases: Preparation, Active Soldering, and Cleanup. In addition, time windows that cannot be assigned to any of these phases with sufficient confidence are assigned to Uncertain Phase. The definitions of these classes are shown in Table 2. Preparation represents preparatory actions before the task, Active Soldering represents the principal soldering actions performed on the circuit board, and Cleanup represents post-task actions related to tidying and completion. Uncertain Phase represents a state that does not sufficiently correspond to any of the three known phases. In practical safety management, such intervals may involve unexpected operations, ambiguity in process transitions, shielding, or operations outside the anticipated workflow. Therefore, they are treated as items requiring verification from the perspective of monitoring work progress and status. For process estimation, time-window features integrate characteristics related to body posture, hand movement, spatial relationships among task-related objects, object recognizability, and gaze-related approximation features. Because the data were acquired at 30 fps, a 30-frame temporal window, corresponding to approximately 1 s, was used to represent short-term body-hand-tool motion patterns. A substantially shorter window would provide less temporal context and could be more sensitive to transient variations, whereas a longer window could contain observations from different phases around phase transitions. Successive windows were shifted by 10 frames (approximately 0.33 s), resulting in approximately 67% overlap. A smaller step would increase redundancy between successive windows, whereas a non-overlapping 30-frame step would reduce the update frequency and provide coarser temporal resolution around phase transitions. The 30-frame window and 10-frame step were therefore adopted as an intermediate setting that provides short-term motion context while maintaining temporal continuity without excessive redundancy. Each time window was assigned the ground-truth phase label that occurred most frequently among the annotated frames within that window. The features calculated for each time window consisted of body features, object spatial-relationship features, object recognizability, and gaze-related approximation features. For the body features, the mean, standard deviation, minimum, maximum, median, interquartile range, intra-window variation, and mean absolute difference were calculated from the three-dimensional coordinate sequences of points such as the nose, shoulders, elbows, wrists, and hand centroids. For both the hand holding the soldering iron and the hand holding the solder wire, the following parameters were also included: elbow angle, wrist velocity, hand center-of-mass velocity, hand opening angle, pinch distance, distance between the two hands, distance from the wrist to the nose, wrist height, wrist depth, and joint-detection failure rate. Object-related features included the minimum three-dimensional distance between the face and the soldering iron, the minimum distance between the soldering iron and the PWB, the minimum distance between the solder wire and the PWB, the minimum distance between the wrist and the soldering iron, the minimum distance between the wrist and the solder wire, and the minimum distance between the tip of the soldering iron and the tip of the solder wire. In addition, the recognizability of the PWB, soldering iron, and solder wire was included as a feature. Gaze-related approximation features represented approximations of gaze direction based on iris position, face orientation, head posture, and the positional relationship with the area around the PWB. During inference, time-window features were first generated from the frame sequences of each trial, and the posterior probabilities of the three known phases were calculated using a random forest (RF) classifier. The phase context used for deviation detection was obtained from the smoothed phase sequence. The smoothed phase was used to reduce short-term fluctuations in phase estimates and to provide a stable process context for subsequent phase-conditioned deviation detection. For each one-second interval, the probabilities of Preparation, Active Soldering, and Cleanup were normalized over the three known phases. The maximum phase probability and the probability margin between the highest and second-highest phases were then calculated. In addition, the ratio of frames or time windows assigned to unavailable or uncertain phase labels was calculated when such labels were present. A one-second interval was assigned to Uncertain Phase when the unavailable-phase ratio exceeded a predefined threshold, when the maximum phase probability was lower than the confidence threshold, or when the probability margin was smaller than the margin threshold. In this study, Uncertain Phase was treated as a review-required interval rather than as a deviation level. Therefore, intervals assigned to Uncertain Phase were excluded from automatic Safe, Caution, and Warning classification. This design prevented low-confidence phase estimates from directly propagating to the phase-conditioned deviation-detection module.

### 2.4. Deviation Detection Based on Soldering Processes

Deviation detection evaluates the extent to which observed posture, movement, object relationships, and gaze-related approximation features depart from the range expected for the current task phase. It is applied only to time windows assigned to one of the three predefined phases: Preparation, Active Soldering, and Cleanup. For time windows assigned to Uncertain Phase, the proposed system does not perform automatic Safe/Caution/Warning classification; instead, it outputs them as review-required intervals. The method therefore detects multivariate deviations from phase-conditioned normal patterns; it does not measure actual danger, injury risk, or the probability of an accident. Features for each 1.0 s interval were calculated using the median of the frame-level features within that interval. The median was used to align the process and deviation sequences on a one-second time axis while suppressing frame-level detection fluctuations and outliers. The examined features included the elbow angles of both arms, wrist velocity, the distance between the face and each wrist, the three-dimensional distances among the face, tools, wrists, and PWB, object visibility and detection reliability, gaze-estimation success and approximation reliability, and head yaw, pitch, and roll. These features were assigned to the categories shown in Table 3 and represent postural load, proximity around the face, tool-workpiece geometry, object visibility, and face-orientation-related observations as quantities directly observable from the sensors. To learn the reference range, feature sequences for 1.0 s intervals obtained from multiple normal trials were used. First, frame-level features were aggregated into one-second features using the median value within each interval. Then, for each feature and each process phase, the one-second median features obtained from the normal trials were collected. The phase-conditioned reference interval was defined using the 5th and 95th percentiles of this distribution as the lower and upper bounds, respectively. The 5th and 95th percentiles were used to define a descriptive central-90% reference envelope in this study. They are not statistical confidence limits, population-level normative bounds, or externally validated safety thresholds. This percentile-based definition reduces the influence of isolated extreme values compared with raw minimum-to-maximum ranges, while preserving the observed distribution of normal operations. Let $x_{j,k}$ denote the observed value of feature *j* in the 1.0 s interval *k*, and let the directionality of feature *j* be ${\mathrm{dir}}_{j}\in \left\{\mathrm{low},\mathrm{high},\mathrm{both}\right\}$. Features such as visibility, detection confidence, gaze-estimation success rate, and distance between the face and the wrist or tool, for which a decrease represents a deviation of interest, are classified as low. Features such as wrist velocity, distance between the tool and the board, and distance between the tip of the soldering iron and the tip of the solder wire, for which an excessive increase represents a deviation of interest, are classified as high. Features such as elbow angle and head angle, for which deviation in either direction is relevant, are classified as both. These classifications were determined on the basis of the operational meaning of each feature.

For each process phase *c*, the directed excess value $o_{j,k}^{\left(c\right)}$ is defined as follows:

(1) $$o_{j,k}^{\left(c\right)}=\left\{\begin{array}{cc} \max\left(L_{j,c}-x_{j,k},\;0\right) & \left({\mathrm{dir}}_{j}=\mathrm{low}\right)\\ \max\left(x_{j,k}-U_{j,c},\;0\right) & \left({\mathrm{dir}}_{j}=\mathrm{high}\right)\\ \max\left(L_{j,c}-x_{j,k},\;x_{j,k}-U_{j,c},\;0\right) & \left({\mathrm{dir}}_{j}=\mathrm{both}\right) \end{array}\right.$$

The feature-level deviation score $d_{j,k}^{\left(c\right)}$ is calculated from the directed excess value for each process phase. When $o_{j,k}^{\left(c\right)}=0$, the deviation score is set to 0. Otherwise, the deviation is normalized using the corresponding feature scale so that features with different units can be integrated. Let $m_{j,c}$ denote the median of feature *j* in the normal reference data for phase *c*. In the implementation used to obtain the reported results, the scale for interval *k* is

(2) $$s_{j,c,k}=\max\left(U_{j,c}-L_{j,c},\;|x_{j,k}|,\;|L_{j,c}|,\;|U_{j,c}|,\;|m_{j,c}|\right).$$

The feature-level deviation score is then

(3) $$d_{j,k}^{\left(c\right)}=\left\{\begin{array}{cc} 0 & \left(o_{j,k}^{\left(c\right)}=0\right)\\ \frac{o_{j,k}^{\left(c\right)}}{s_{j,c,k}} & (o_{j,k}^{\left(c\right)}>0). \end{array}\right.$$

The implementation computes the scale as the maximum of an overflow-tolerance term, the reference-support width $U_{j,c}-L_{j,c}$, and the largest absolute magnitude among the current observation, the lower and upper reference bounds, and the normal median. For the percentile-based reference construction used for the reported evaluation, the overflow-tolerance term was set to 0, which yields Equation (2). Because the current observation $x_{j,k}$ is included in the magnitude term, the normalization scale depends on both the phase *c* and the evaluated interval *k*. This magnitude-adaptive engineering normalization was used to reduce domination of the integrated score by features with different physical units and absolute numerical scales; it should not be interpreted as a learned safety calibration or as a population-level risk scale.

The category score $p_{g,k}^{\left(c\right)}$ is defined as the average of the deviation scores belonging to category *g* for phase *c*. Let $G_{c}$ denote the set of categories valid for process phase *c*. The phase-specific deviation score $D_{c}\left(k\right)$ is then calculated as follows:

(4) $$D_{c}\left(k\right)=\frac{1}{|G_{c}|}\sum_{g\in G_{c}}p_{g,k}^{\left(c\right)}.$$

The final score is not calculated from a single hard phase label. Instead, deviation scores are calculated for all three predefined phases and integrated using the smoothed phase probabilities. Let $q_{c}\left(k\right)$ denote the smoothed probability of process phase *c* at interval *k*. The final deviation score $R_{k}$ is defined as

(5) $$R_{k}=\sum_{c\in \{\mathrm{Preparation},\mathrm{Active}\;\mathrm{Soldering},\mathrm{Cleanup}\}}q_{c}\left(k\right)D_{c}\left(k\right).$$

With this design, an unsafe-like state is described in terms of how it deviates from the normal pattern of each process phase across multiple characteristics. Therefore, in addition to identifying when a deviation occurred, it is possible to trace which body part or functional category contributed to the assessment.

## 3. Evaluation

In this section, we evaluated whether the proposed system could estimate the progression of seated manual soldering and identify localized deviations associated with Simulated Unsafe-like Movements.

### 3.1. Experimental Scenario

The study comprised ten 60 s soldering sessions obtained from five participants: five normal sessions and five sessions containing Simulated Unsafe-like Movements. In this manuscript, one recorded soldering session corresponds to one 60 s trial. The participants were recruited from students at the authors’ institution and were purposively selected to include variation in body size. Their heights ranged from 1.55 to 1.76 m, and their soldering experience ranged from 0.2 to 5.2 years. The right hand was standardized as the tool-holding hand and the left hand as the solder-wire-holding hand in the experimental protocol, rather than assuming that the right hand was dominant for every participant. In both conditions, the participants performed seated manual soldering and completed Preparation, Active Soldering, and Cleanup within 60 s.

Actual accidents or intentional contact with a hot soldering iron tip were not induced for ethical and safety reasons. The scripted movements were deliberately performed under controlled and safe conditions to create deviations from the normal task pattern, including changes in the relationship between the face and the tool-holding hand, unnatural upper-limb posture, and actions that did not match the learned phase patterns. The normal trials represented standard task execution, whereas the scripted movements were inserted into parts of the corresponding phases while preserving the overall process sequence.

Frame-level phase labels were assigned from the recorded videos according to predefined operational criteria based on observable task events. Preparation extended from the beginning of the task until the soldering iron first intentionally approached the joining area on the PWB. Active Soldering extended from the initiation of the joining operation until the soldering iron and solder wire were withdrawn from the joining area after the final operation. Cleanup then extended from the beginning of tool and PWB organization until the tools were returned to their designated positions and the participant assumed the task-completion posture. The experimental-state labels were Safe, Caution, and Warning; Warning was assigned to frames during which a scripted simulated unsafe-like movement was being performed. Caution was assigned from the first frame in which a preparatory movement toward the scripted unsafe-like movement became visually observable until the frame immediately preceding the onset of Warning. To assess annotation reliability, the first author (K.W.) and a member of the same laboratory independently assigned the phase and experimental-state labels to the same video data on a frame-by-frame basis according to the predefined guidelines. The two annotators performed the labeling without access to each other’s annotations. The agreement statistics were calculated from these independently assigned labels before adjudication. The frame-wise agreement was 92.50% for the phase labels and 94.05% for the experimental-state labels, with Cohen’s kappa coefficients of 0.888 and 0.885, respectively. For the ordinal experimental-state labels, the linearly weighted Cohen’s kappa was 0.935. After the reliability analysis, disagreements were resolved through discussion between the two annotators, and the resulting consensus labels were used as the final ground truth. This procedure evaluates inter-annotator consistency within the present study, but it should not be interpreted as validation by multiple independent occupational-safety experts because both annotators were members of the same laboratory.

For deviation detection, a participant-excluded evaluation procedure was applied separately to each participant. When a participant was evaluated, the phase-conditioned reference intervals were constructed exclusively from the normal trials of the other four participants; the evaluated participant’s own normal trial was never included in the reference data. Likewise, the phase probabilities supplied to the deviation-detection stage were out-of-fold predictions generated by a phase-estimation model trained without any data from the participant being evaluated. The five trials containing Simulated Unsafe-like Movements, one from each participant, were divided at the participant level: the simulated unsafe-like trials from two participants were used for threshold development, whereas the simulated unsafe-like trials from the other three participants were reserved for final held-out evaluation. The same participant-exclusion rule for both reference-interval construction and phase-probability generation was applied to the threshold-development trials and to the final evaluation trials.

**Figure 3:**
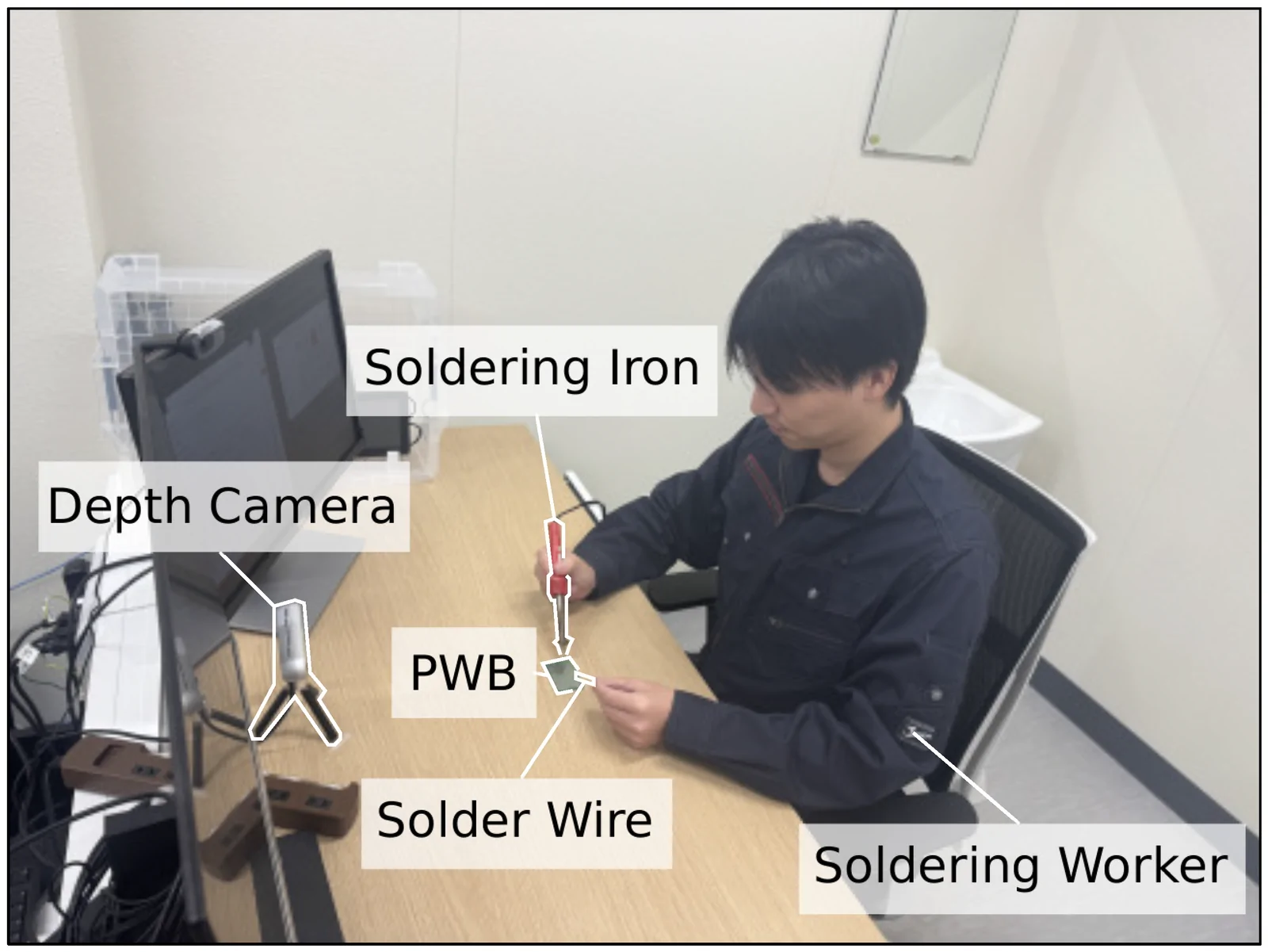
Experimental environment.

Figure 3 shows the experimental environment. The participant sat in front of the depth camera so that the face, upper body, both hands, and tabletop workspace could be observed simultaneously. The participant held the soldering iron in the right hand and the solder wire in the left hand. The sensing configuration was kept the same across all trials. For each frame, the system estimated three-dimensional body landmarks, hand- and face-related features, and the positions of the PWB, soldering iron, and solder wire. It then output features related to posture, movement, object visibility, and the three-dimensional relationships among the operator, tools, and workpiece. The evaluation examined the continuity of the estimated process sequence, changes in elbow angle and distance from the face, the stability of gaze-related approximation features, changes in object visibility, and correspondence with the integrated deviation score. In addition, the classifier and baseline comparisons quantitatively evaluated process-phase estimation accuracy, detection accuracy for Simulated Unsafe-like Movements, and classification performance for each state.

**Figure 4:**
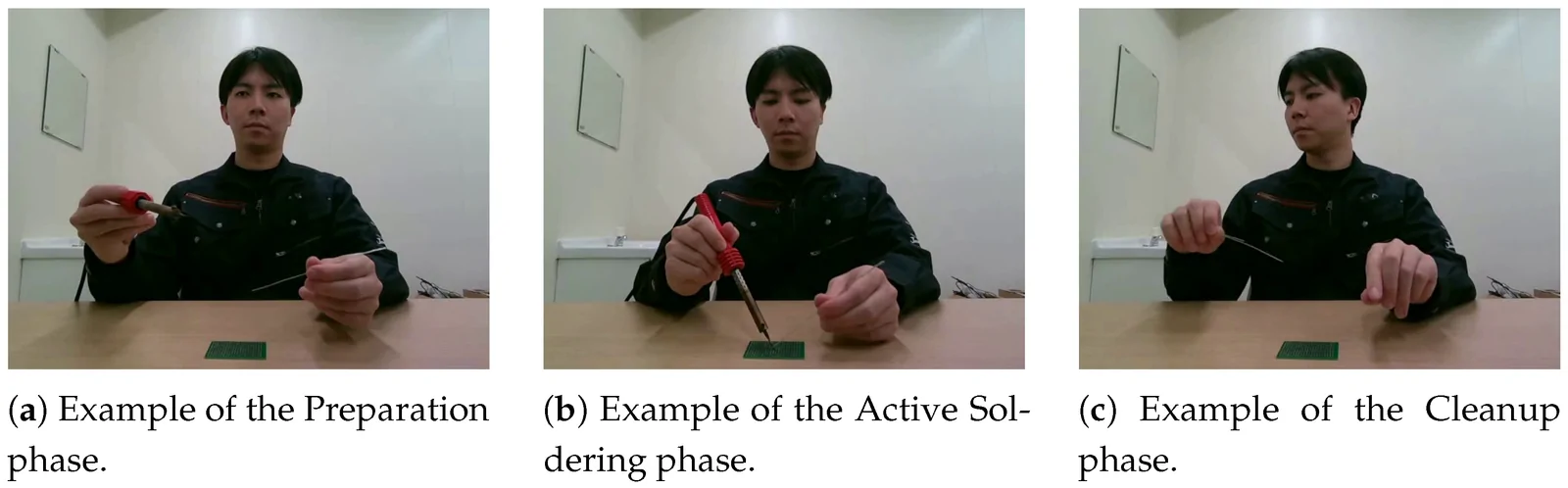
Examples of each phase in a typical normal trial.

**Figure 5:**
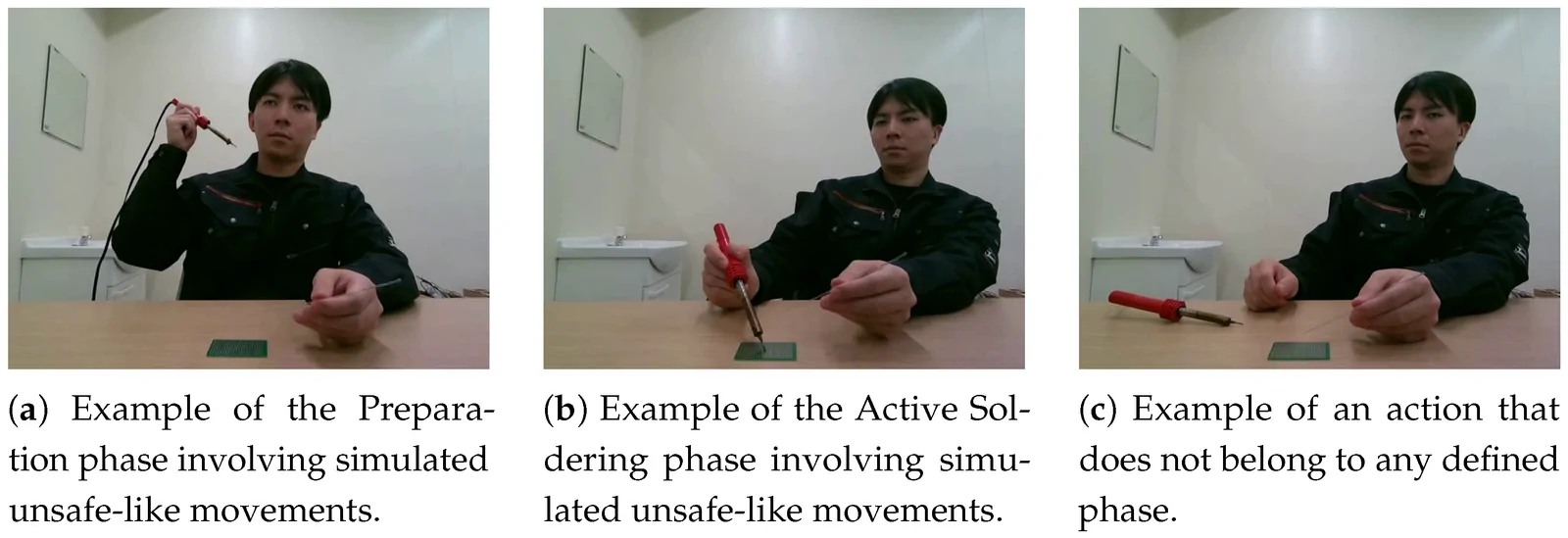
Examples of simulated unsafe-like movements and actions that do not belong to any defined phase.

In a typical normal trial, as shown in Figure 4, seated manual soldering was performed in the order of Preparation, Active Soldering, and Cleanup. In the Preparation phase, the PWB, solder wire, and soldering iron were placed in their working positions. In the Active Soldering phase, a series of soldering operations, including approach, heating, material supply, and withdrawal, were performed on the board. In the Cleanup phase, the used tools and the PWB were returned to their designated locations, and the task was completed. In the trials involving Simulated Unsafe-like Movements, movements simulating unsafe situations were intentionally inserted into parts of the Preparation, Active Soldering, and Cleanup phases while preserving the overall process sequence. Figure 5 shows examples of these movements. In this trial, the Simulated Unsafe-like Movements were designed to appear mainly around 20 s and 30 s. In addition, these movements could result in Uncertain Phase segments in intervals where the process phase became ambiguous. In the Preparation phase, the trial included movements such as changing the positional relationship between the hand holding the tool and the face from that observed during normal work. In the Active Soldering phase, the trial included movements such as continuing work while maintaining an unnatural arm posture, as well as movements that were not observed in normal trials. Furthermore, in the Cleanup phase, an action that did not belong to any of the Preparation, Active Soldering, or Cleanup phases was inserted to create a state that did not match the learned process phase patterns. Such an action does not clearly correspond to a specific phase and may therefore be assigned to Uncertain Phase. The thresholds used as criteria for Caution and Warning were derived only from the two participant-level development trials and were fixed before evaluation of the remaining three simulated unsafe-like trials. For threshold development, the same operational labels defined above were used: Warning corresponded to the scripted simulated unsafe-like movement itself, whereas Caution covered the visually observable preparatory movement immediately preceding its onset. Table 4 presents the YOLO performance on the participant-disjoint test set. The class-wise rows report segmentation-mask metrics. For the bounding boxes overall, the precision was 0.863, the recall was 0.828, the mean average precision (mAP)@0.5 was 0.866, and the mAP@0.5:0.95 was 0.704. For the segmentation masks overall, the precision, recall, mAP@0.5, mAP@0.5:0.95, and mean semantic intersection over union (IoU) were 0.810, 0.733, 0.732, 0.591, and 0.687, respectively. At the class level, the PWB and soldering iron showed high segmentation-mask performance, whereas the solder wire showed substantially lower precision, recall, mAP, and semantic IoU values, indicating that the solder wire was the most difficult of the three task-related objects to segment reliably.

### 3.2. Comparison Results of Process Phase Estimation Models

The process-phase estimator used in the proposed system was implemented using an RF classifier. Support vector machine (SVM)-based classifiers with a radial basis function (RBF) kernel (SVM-RBF) and a linear kernel (SVM-linear) were evaluated as comparison methods. RF with Viterbi-based temporal smoothing was additionally evaluated to examine the effectiveness of temporal consistency. RF was selected because the present dataset consisted of engineered tabular multimodal features from a small number of sessions. RF can model nonlinear feature relationships, provide class probabilities for soft phase integration, and permit feature-level interpretation. All methods were evaluated using the same multimodal feature dataset. Five-fold subject-wise leave-one-participant-out validation was implemented using GroupKFold, with the participant identifier as the group identifier. In each fold, all recording sessions belonging to one participant were held out for testing, while all sessions from the remaining four participants were used for training. Thus, the two sessions from the same participant were never divided between the training and test sets. All overlapping 30-frame windows generated with a 10-frame step from the same participant were assigned to the same fold. Therefore, neighboring or overlapping windows from the same participant did not occur in both the training and test portions of a fold, avoiding direct data leakage due to window overlap. Each 30-frame window was assigned the ground-truth phase that occurred most frequently among the annotated frames within that window. Windows for which a valid ground-truth phase could not be assigned were excluded from model training and evaluation. For RF, the number of trees was set to 400, and the minimum number of samples required at a leaf node was set to 2. The evaluation targeted the three process phases of Preparation, Active Soldering, and Cleanup. Accuracy, Macro-F1, Weighted-F1, and the F1-score for each phase were calculated. These evaluation metrics were treated as normalized scores ranging from 0.0 to 1.0. Accuracy represents the ratio of correctly classified samples to all samples. Macro-F1 is the unweighted average of the F1-scores for each phase and is less affected by differences in the number of samples among phases. In contrast, Weighted-F1 is the F1-score weighted by the number of samples in each phase and was used to evaluate the overall phase estimation performance. The F1-score for each phase was calculated to individually examine the estimation performance for each process phase. In RF with Viterbi-based temporal smoothing, the output sequence of RF was smoothed by considering the chronological structure of the soldering process. The ordinary workflow proceeds from Preparation to Active Soldering and then to Cleanup; therefore, temporal smoothing was introduced to suppress short-term fluctuations in the estimated phase labels. The parameters of the Viterbi-based temporal smoothing were determined using the training and validation data and were not tuned on the test data. In this evaluation, Viterbi smoothing was applied as an offline temporal post-processing step. Table 5 shows the comparison results of the process phase estimation methods. RF with Viterbi-based temporal smoothing achieved the highest overall performance, with an Accuracy of 0.729, a Macro-F1 of 0.729, and a Weighted-F1 of 0.736. Compared with RF without temporal smoothing, the Accuracy, Macro-F1, and Weighted-F1 improved by 0.086, 0.086, and 0.083, respectively. RF without temporal smoothing achieved an Accuracy of 0.643, a Macro-F1 of 0.643, and a Weighted-F1 of 0.653. SVM-RBF achieved an Accuracy of 0.608 and a Macro-F1 of 0.608, whereas SVM-linear achieved an Accuracy of 0.598 and a Macro-F1 of 0.593. These results indicate that RF outperformed the SVM-based classifiers in terms of overall phase estimation performance. For each process phase, RF with Viterbi-based temporal smoothing achieved the highest F1-scores for all three phases. In particular, the F1-scores for Preparation and Cleanup improved from 0.535 to 0.674 and from 0.542 to 0.656, respectively, compared with RF without temporal smoothing. In contrast, the F1-score for Active Soldering changed only slightly from 0.850 to 0.857. This result suggests that RF already classified Active Soldering relatively accurately, whereas temporal smoothing was especially effective for stabilizing the estimation of Preparation and Cleanup, which are more likely to be affected by ambiguous transition intervals. Therefore, RF with Viterbi-based temporal smoothing was adopted as the process phase estimator in the proposed system because it achieved the highest overall performance and improved the estimation stability of the process phases. Table 6 shows the phase-wise performance of RF with Viterbi smoothing. Active Soldering achieved the highest F1-score of 0.857 and the lowest FPR of 0.018. The F1-scores for Preparation and Cleanup were 0.674 and 0.656, respectively, indicating that the main difficulty was distinguishing these two relatively low-motion, tool-placement phases, whereas Active Soldering was comparatively well distinguished.

### 3.3. Comparison Results of the Proposed and Baseline Deviation-Detection Methods

To evaluate the proposed method, two baseline methods were used as ablation-style reference configurations that facilitate the interpretation of the main design elements of the framework. Baseline A did not use phase-conditioned references and instead constructed a single global reference range from the remaining participants’ normal trials, providing a reference for examining the role of phase context. Baseline B represented a simpler single-feature heuristic in which the decision was based on the largest deviation among selected individual features rather than integrated multifeature deviations. For each participant under evaluation, all reference ranges used by the proposed method and the baselines were constructed exclusively from the normal trials of the remaining four participants. For the proposed method, the phase probabilities were generated out-of-fold by a phase-estimation model trained without any data from the participant under evaluation. The five simulated unsafe-like trials were separated at the participant level: the simulated unsafe-like trials from two participants were used for threshold development, whereas the simulated unsafe-like trials from the other three participants were used only for final held-out evaluation. Each feature acquired at 30 fps was aggregated every second by calculating the median value of the frame-level values within each one-second interval. The one-second median features obtained from the normal trials were then collected for each process phase. The 5th and 95th percentiles of these distributions were used as the lower and upper bounds of the phase-dependent reference range, respectively. The 5th and 95th percentiles were used to define a descriptive central-90% reference envelope in this study. They are not statistical confidence limits, population-level normative bounds, or externally validated safety thresholds. For the proposed method, the final deviation score was calculated by integrating the deviation scores for each process phase according to the smoothed phase probabilities, thereby reducing direct dependence on a single hard phase label. The Caution and Warning score thresholds were determined using only the two participant-level development trials. Intervals routed to review were excluded from this fitting step because review is not a deviation-level label. Candidate thresholds were constructed from the ordered unique finite deviation scores observed in the development data. Every candidate pair satisfying $T_{\mathrm{Caution}}\leq T_{\mathrm{Warning}}$ was evaluated on the development data. Safe was treated as the negative class and Caution plus Warning as the positive class for the binary metrics. Candidate threshold pairs were ranked lexicographically, with Binary-F1 as the primary criterion, followed by higher Warning recall, lower FPR, higher Macro-F1, and higher Accuracy for deterministic tie-breaking. No held-out evaluation data were used for threshold selection. The resulting thresholds were $T_{\mathrm{Caution}}=0.0108$ and $T_{\mathrm{Warning}}=0.0194$, and these values were fixed before application to the three held-out evaluation trials. For intervals not assigned to Uncertain Phase, deviation scores below 0.0108 were classified as Safe, scores from 0.0108 to below 0.0194 as Caution, and scores of 0.0194 or higher as Warning. Uncertain Phase was not treated as a deviation label; it was separated from the automatically assessed seconds as an interval requiring review because the process context could not be estimated with sufficient confidence. The confidence, margin, and unavailable-phase ratio thresholds were selected separately from the Caution/Warning score thresholds. Candidate review-routing configurations were evaluated using only the development trials and ranked lexicographically. Binary-F1 was used as the primary criterion, followed by higher Warning recall, lower review rate, lower FPR, higher Macro-F1, and higher Accuracy. The selected configuration was fixed before application to the held-out evaluation trials. The selected values were 0.35, 0.05, and 0.75, respectively. These thresholds routed 11.1% of the evaluation intervals to review while retaining automatic coverage of 88.9%. Baseline A and Baseline B natively assigned a class to all 180 s and did not use the proposed review gate. For a matched-subset comparison, however, the same 20 s routed to review by the proposed method were excluded from all three methods, leaving an identical 160 s evaluation subset for calculation of Accuracy, Macro-F1, Binary-F1, FPR, FNR, and Warning recall. In this section, Accuracy represents the correct classification rate for all evaluated samples, Macro-F1 is the simple average of the F1-scores for each class, and Binary-F1 is the F1-score calculated by treating Safe as the negative class and Caution and Warning as the positive class. The false-positive rate (FPR) represents the proportion of normal states incorrectly classified as unsafe-like deviations. The false-negative rate (FNR) represents the proportion of unsafe-like deviations incorrectly classified as normal states. Warning recall represents the proportion of ground-truth Warning intervals correctly classified as Warning. All these metrics were treated as normalized scores ranging from 0 to 1.

Table 7 shows the baseline comparison results for deviation detection. The proposed method assigned 20 s of the 180 s evaluation data to Uncertain Phase, corresponding to a review rate of 0.111. Accordingly, the classification metrics reported in Table 7 for the proposed method and both baselines were calculated on the same remaining 160 s. The review-required intervals consisted of 11 s with the ground-truth label Safe, 1 s with Caution, and 8 s with Warning. As a result, on the common matched 160 s subset, the proposed method achieved an Accuracy of 0.750, a Macro-F1 of 0.511, and a Binary-F1 of 0.772, the highest values among the three evaluated configurations on that subset. In particular, on the common matched 160 s subset, for Binary-F1, where Safe was treated as the negative class and Caution and Warning were treated as the positive class, the proposed method achieved 0.772, compared with 0.565 for Baseline A and 0.635 for Baseline B. Baseline A showed an FPR of 0.216 and an FNR of 0.453, indicating that the phase-unaware global reference range tended to miss unsafe-like states by classifying them as Safe. In contrast, the proposed method reduced the FNR to 0.082, suggesting that the use of phase-conditioned reference ranges and phase-conditioned scoring helped suppress missed detections of deviations associated with simulated unsafe-like movements. On the other hand, Baseline B showed an FNR of 0.156 and a Warning recall of 0.696, indicating relatively high sensitivity to unsafe-like states. However, its FPR was as high as 0.448, indicating a tendency to over-detect Safe intervals as Caution or Warning. On the common matched subset, the proposed method achieved a higher Warning recall of 0.942 and a lower FNR of 0.082 than both baselines while keeping the FPR at 0.283. Although the FPR of the proposed method was higher than that of Baseline A, it was lower than that of Baseline B, and the proposed method achieved the highest Binary-F1 on the common matched subset. This result indicates that separating intervals with unclear process context from automatic judgment and assigning them to review functioned as intended, rather than immediately converting such intervals into deviation decisions. Therefore, the Uncertain Phase should not be interpreted as directly indicating an actual process anomaly, but rather as auxiliary information indicating intervals in which the observed features did not sufficiently match the learned process phase patterns. The 20 s exclusion shown for the baselines in Table 7 is therefore a common matched-subset exclusion used only for fair metric comparison and does not represent native abstention by either baseline. The corresponding 11.1% review rate is a property of the proposed framework only. Because more than one component was simplified in Baseline B, the comparison is interpreted as an ablation-style analysis rather than a strict component-wise ablation study.

On the common matched 160 s subset, the proposed method achieved the highest Accuracy, Macro-F1, and Binary-F1 among the three evaluated configurations. These rankings are conditional on the matched subset selected by the proposed review gate and therefore do not establish superiority at native 100% automatic coverage. The result is consistent with the ability of the proposed method to account for phase-dependent variations in normal posture, hand position, tool position, and work-object visibility, using phase-conditioned reference ranges and integrating phase-specific deviation scores according to the smoothed phase probabilities. In Baseline A, which did not consider process phases, the global reference range tended to miss deviations associated with simulated unsafe-like movements by classifying them as Safe. In contrast, Baseline B was sensitive to deviations in a single feature but also caused many over-detections in Safe intervals. Therefore, on the matched evaluation subset, the proposed configuration showed a more favorable balance between detecting unsafe-like states and suppressing excessive over-detection than the two evaluated baselines; this comparison should not be extrapolated to untested native 100% coverage. The phase-wise performance of the proposed method is summarized in Table 8. For the phase-wise evaluation, Accuracy and FPR were calculated in a one-vs-rest manner for each phase. As shown in Table 8, Active Soldering achieved the highest Binary-F1 of 0.931. For Preparation, the Binary-F1 was 0.600, with a Recall of 1.000 and an FPR of 0.157. In contrast, Cleanup showed a Binary-F1 of 0.250 and an FPR of 0.419. Although all three positive seconds in Cleanup were detected, 18 Safe seconds were classified as unsafe-like, resulting in a Precision of 0.143.

### 3.4. Experimental Results for Normal Trials

**Figure 6:**
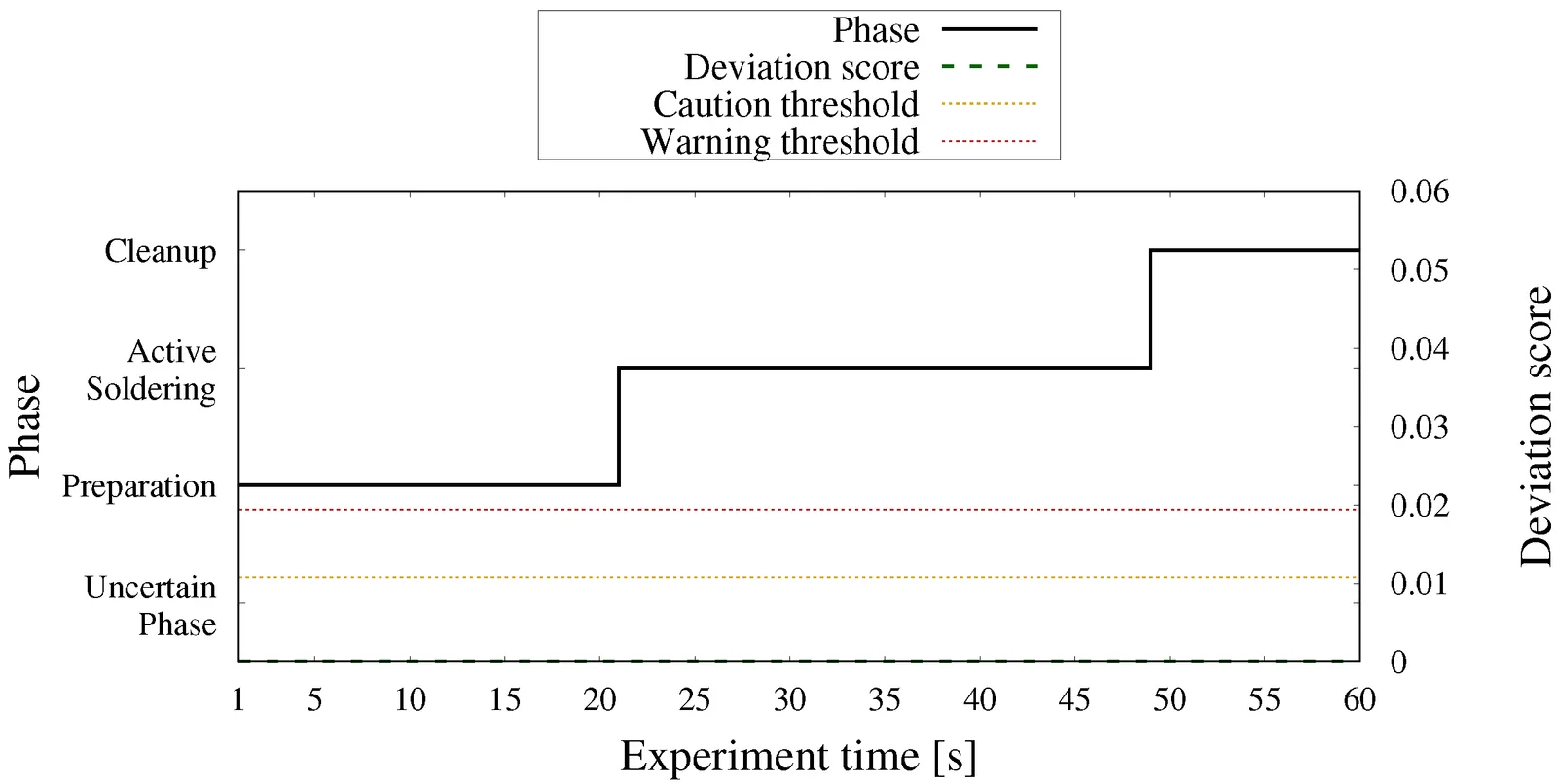
Estimated phases and deviation scores for a normal trial.

**Figure 7:**
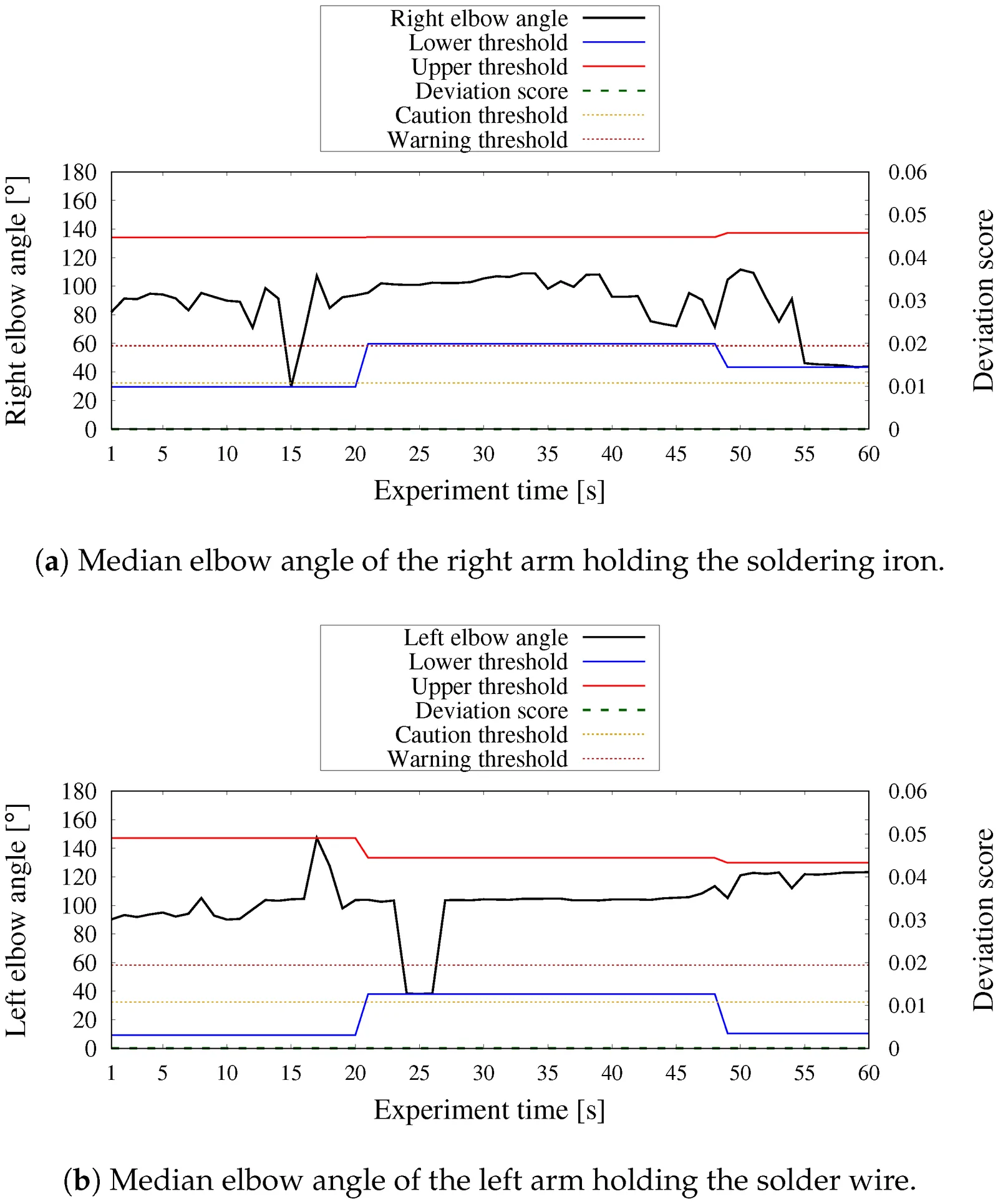
Temporal changes in elbow angle during a typical normal trial.

**Figure 8:**
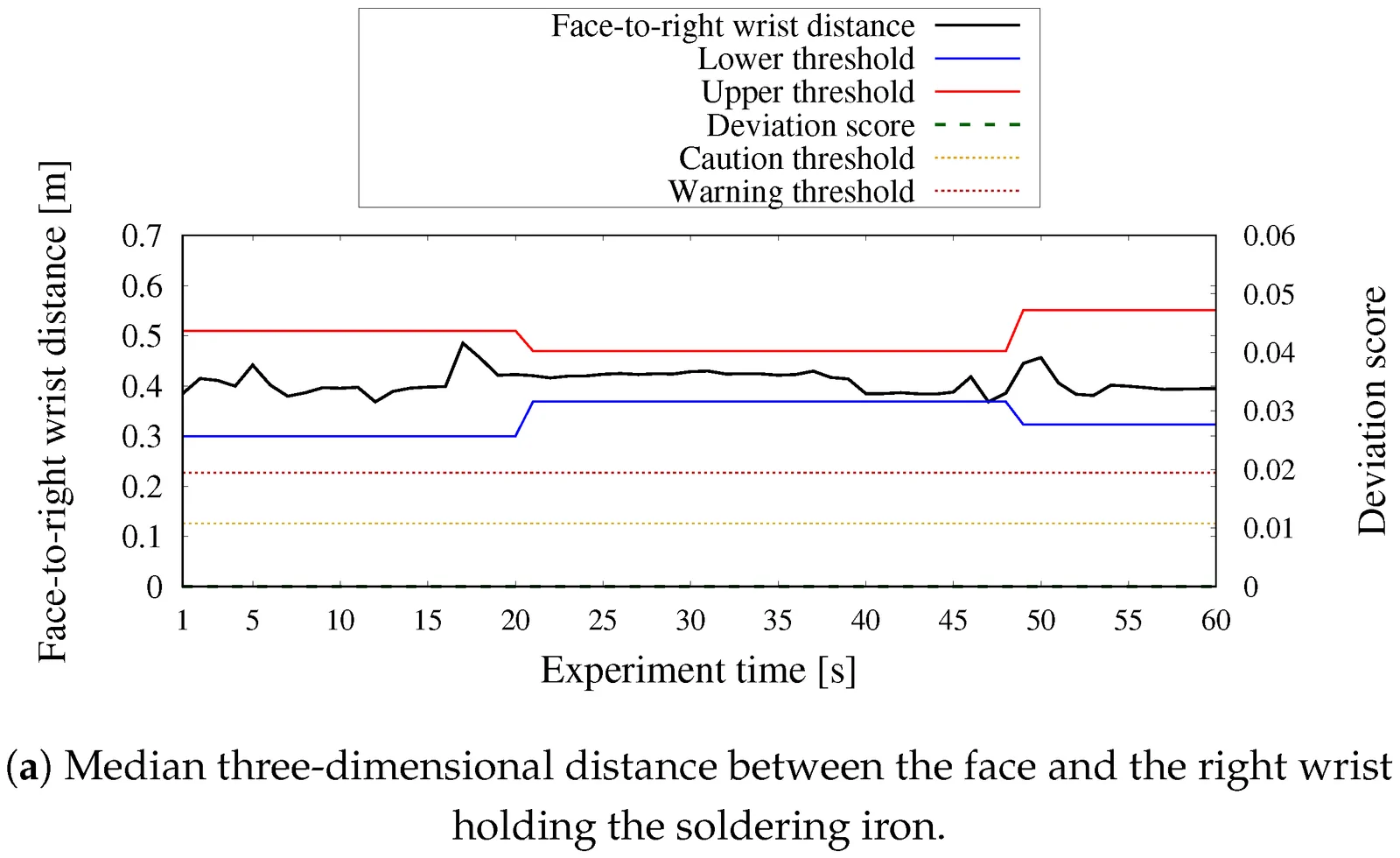

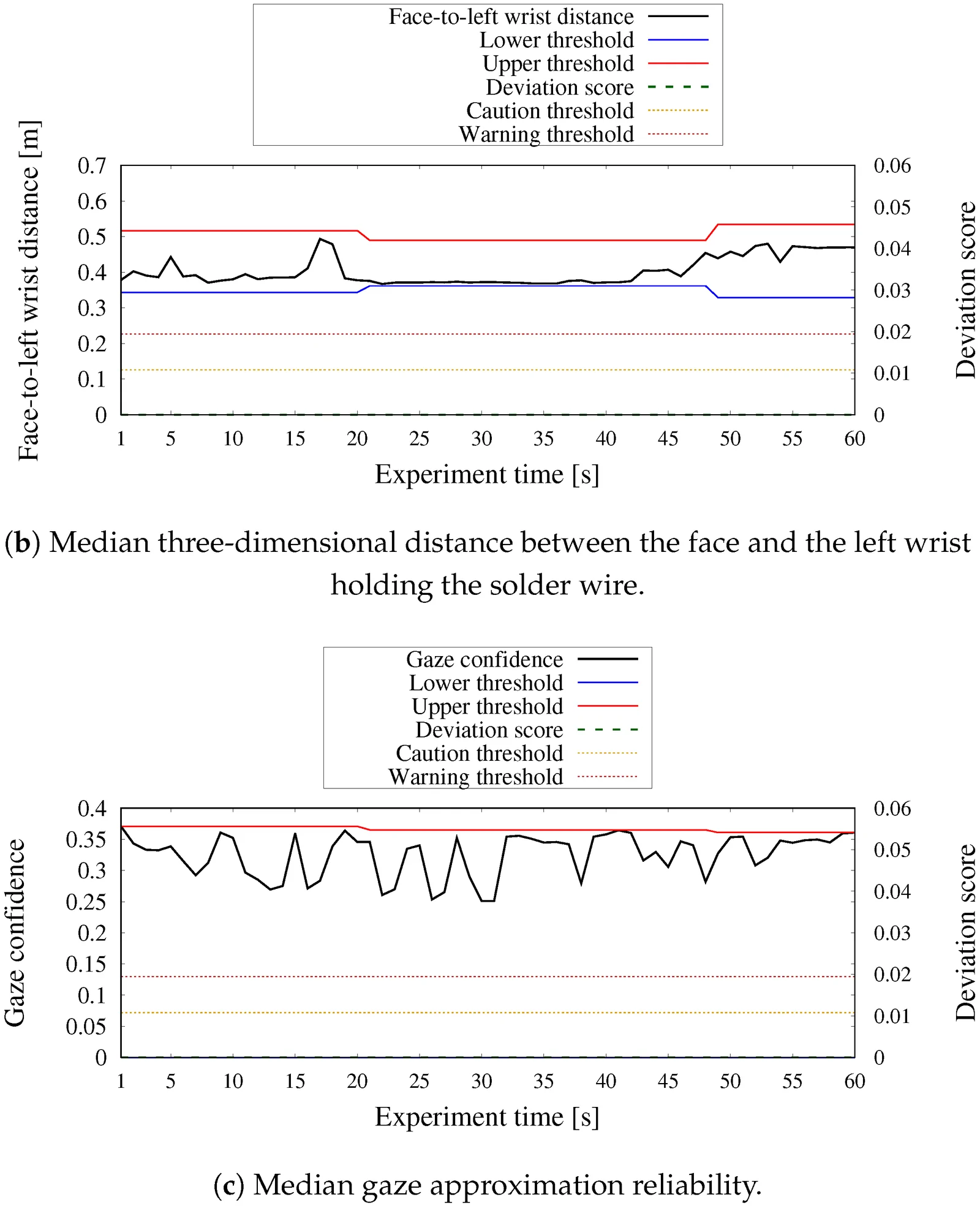
Temporal changes in face-to-wrist distance and gaze-related approximation features during a typical normal trial.

**Figure 9:**
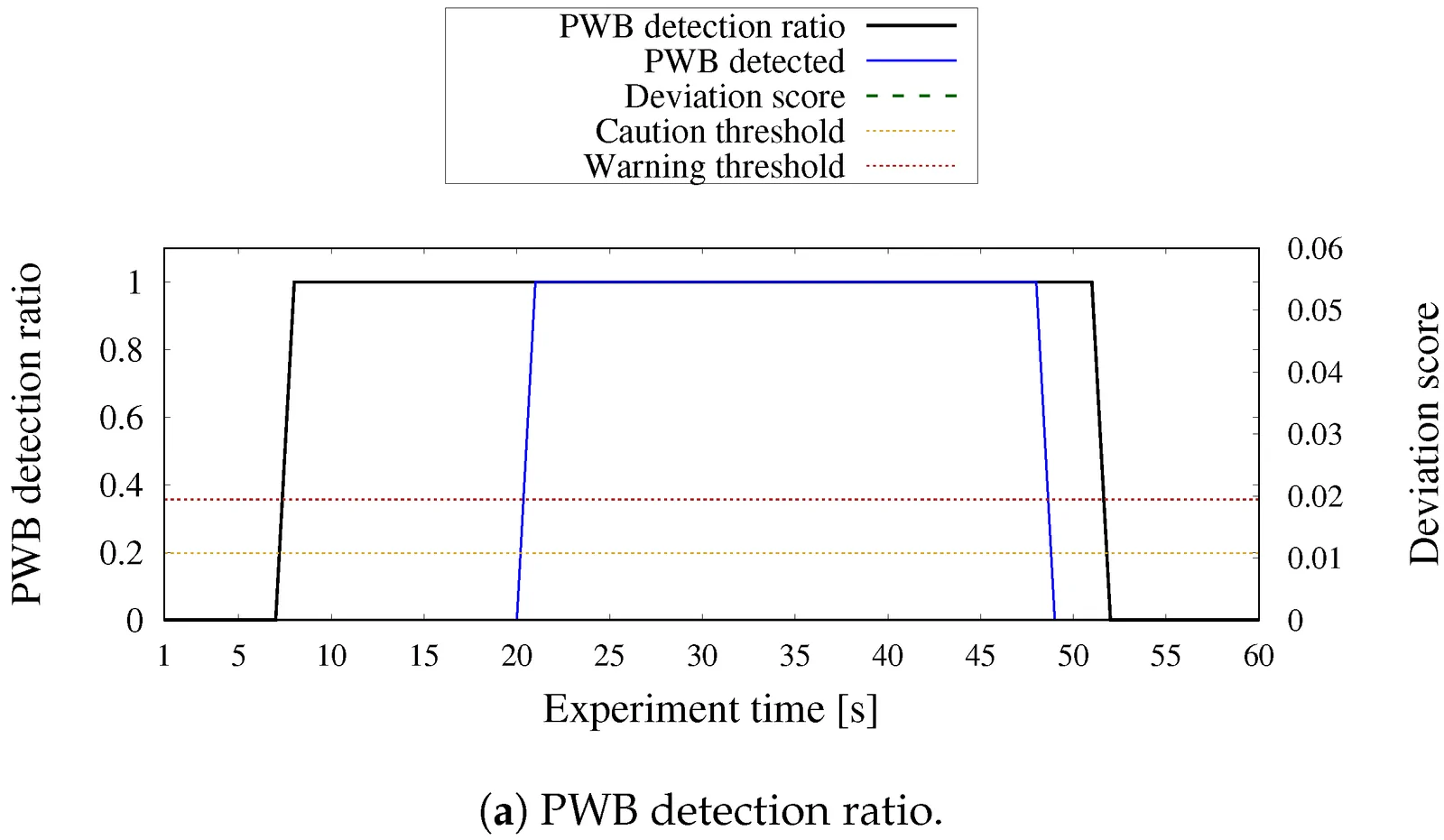

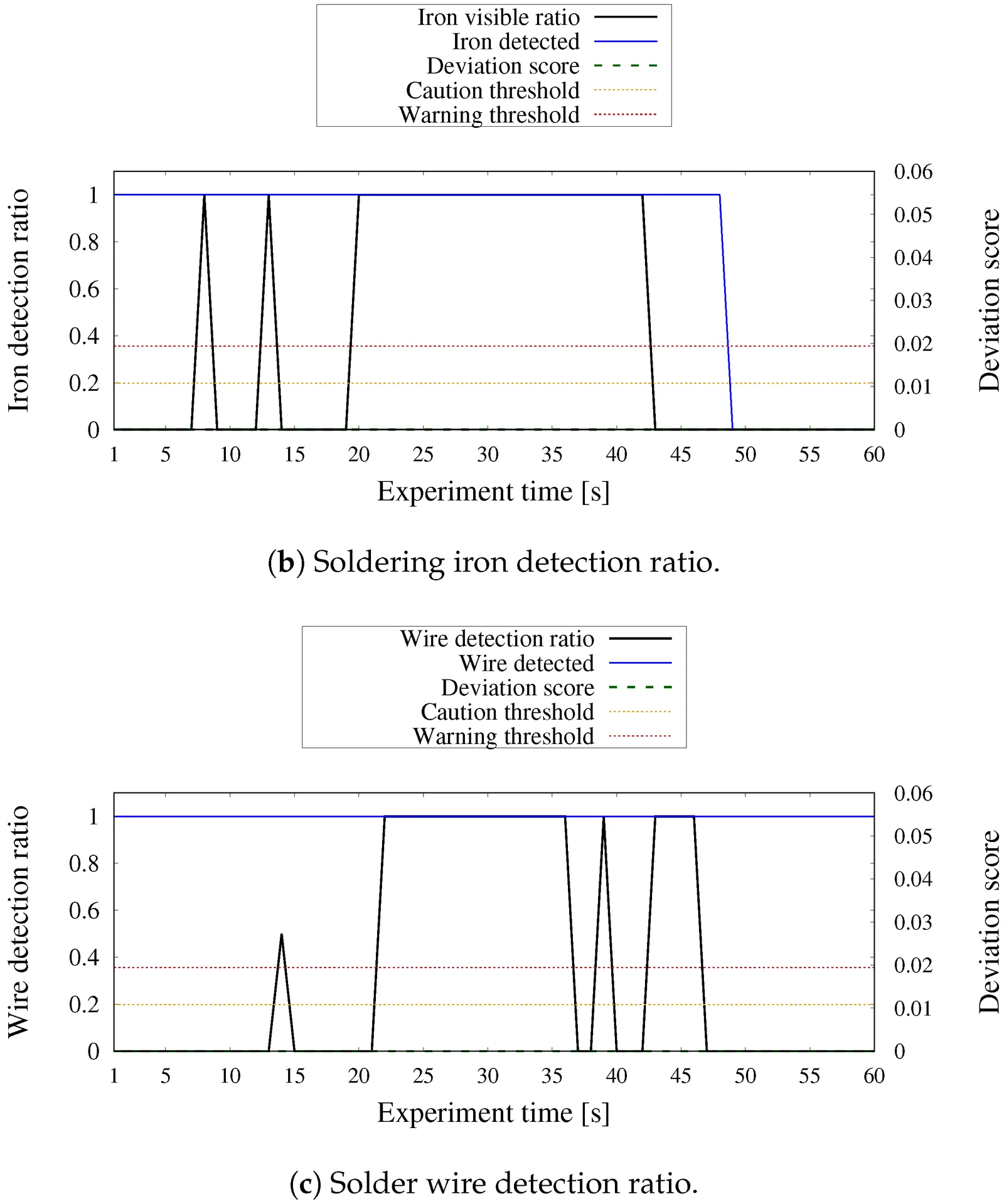
Temporal changes in object detection ratio during a normal trial.

Figure 6 shows the estimated process phases and the deviation score for a normal trial. In this trial, the deviation level was classified as Safe throughout the entire 60 s interval, and the maximum, mean, and median deviation scores were all 0.0. The estimated phase durations were 20 s for Preparation, 28 s for Active Soldering, and 12 s for Cleanup. No Uncertain Phase segment was observed, and the process progressed continuously from Preparation through Active Soldering to Cleanup. These results indicate that the process sequence in the normal trial was estimated consistently and that the observed feature combinations did not deviate from the reference range for each phase. The deviation score remained low throughout the trial, indicating that the observed state was consistent with the normal condition of the corresponding process phase. As shown in Figure 7, the elbow angles of both arms remained within their phase-dependent reference ranges. The median elbow angle of the right arm holding the soldering iron ranged from $29.4^{\circ }$ to $111.6^{\circ }$, and no interval was observed in which the value fell outside the reference range. The median elbow angle of the left arm holding the solder wire ranged from $38.0^{\circ }$ to $147.2^{\circ }$, and it also remained within the reference range throughout the trial. These results indicate that the upper-limb posture was maintained without abrupt collapse or excessive deviation during the normal soldering task. As shown in Figure 8, the three-dimensional distances between the face and both wrists, as well as the gaze approximation reliability, also remained within their reference ranges. The distance between the face and the right wrist ranged from 0.368 m to 0.485 m, whereas the distance between the face and the left wrist ranged from 0.367 m to 0.493 m. In both cases, no interval was detected outside the reference range. Furthermore, the gaze approximation reliability varied from 0.251 to 0.371, and no substantial decrease was observed during the trial. These results suggest that the tool-holding hand did not approach the face excessively and that the relationship among face orientation, head posture, and the work object remained consistent with the normal working state. Temporal changes in the object detection ratio are shown in Figure 9. The detection ratios of the PWB, soldering iron, and solder wire varied between 0 and 1 according to the progress of the task. The mean detection ratios were 0.733 for the PWB, 0.417 for the soldering iron, and 0.342 for the solder wire. Although the detection ratios changed over time depending on the visibility of each object, no increase in the deviation score was observed for the object detection ratio features. Therefore, the temporal changes in object visibility were regarded as normal variations associated with the soldering process and did not contribute to an increase in the deviation score. Based on the above results, the normal trial showed a consistent process sequence, stable upper-limb posture, appropriate face-to-wrist distances, stable gaze-related approximation features, and object detection ratio features that did not increase the deviation score. These findings confirm that the proposed method classified the entire normal trial as Safe and that no feature-level deviation or Uncertain Phase segment was detected in this case.

### 3.5. Experimental Results of Trials Involving Simulated Unsafe-like Movements

**Figure 10:**
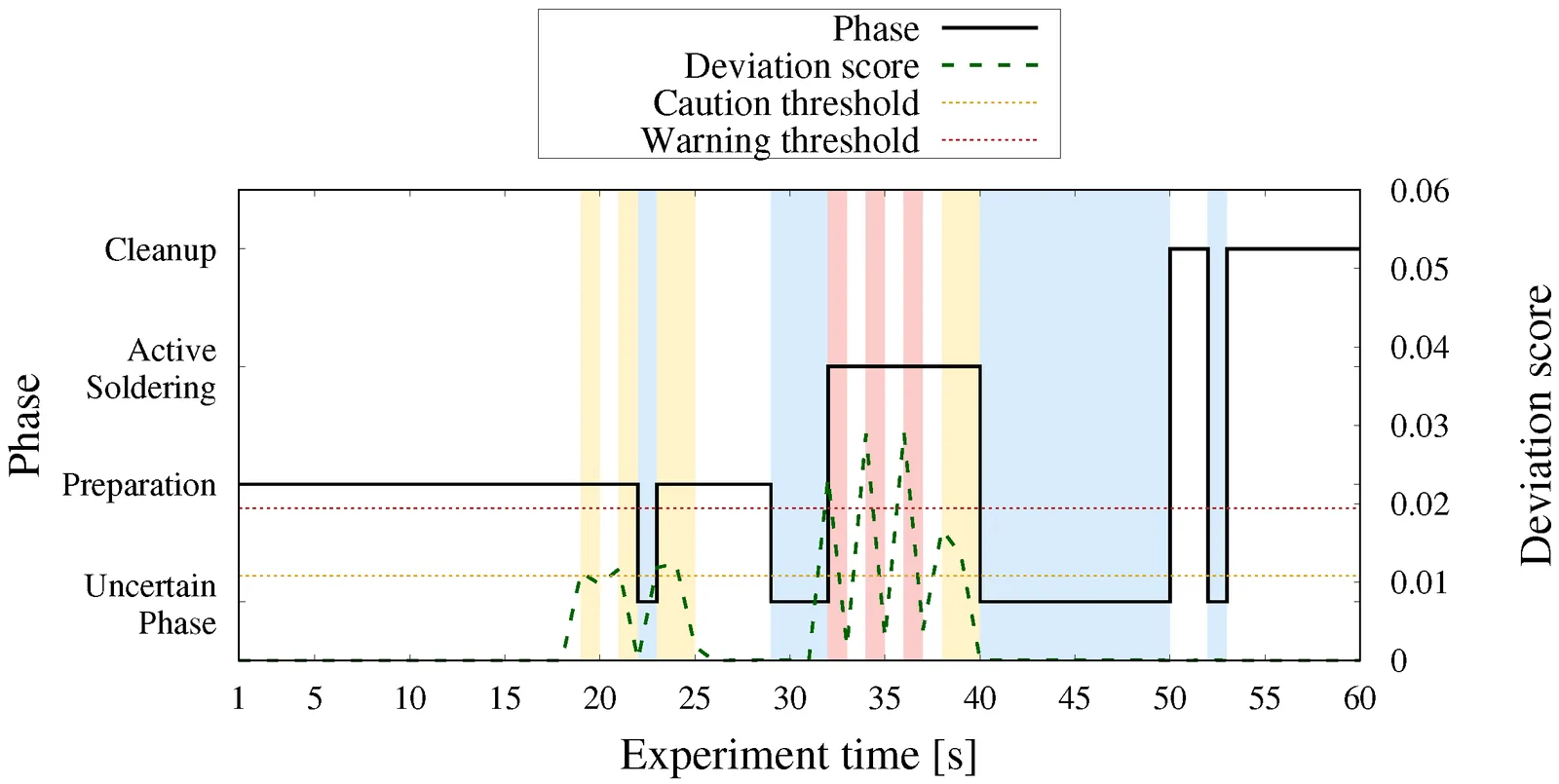
Estimated phases and deviation scores for a trial involving simulated unsafe-like movements.

**Figure 11:**
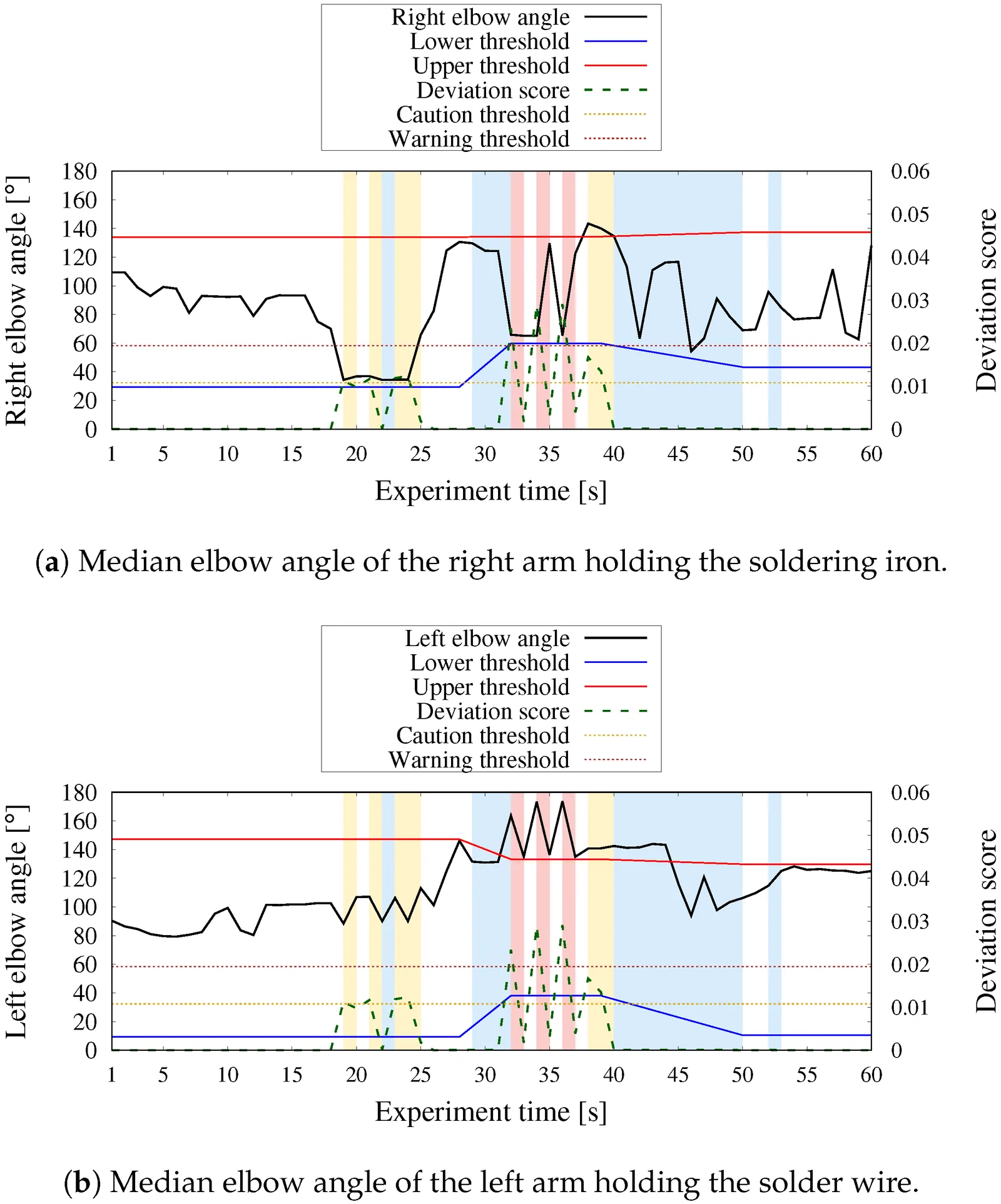
Temporal changes in elbow angle during a trial involving simulated unsafe-like movements.

**Figure 12:**
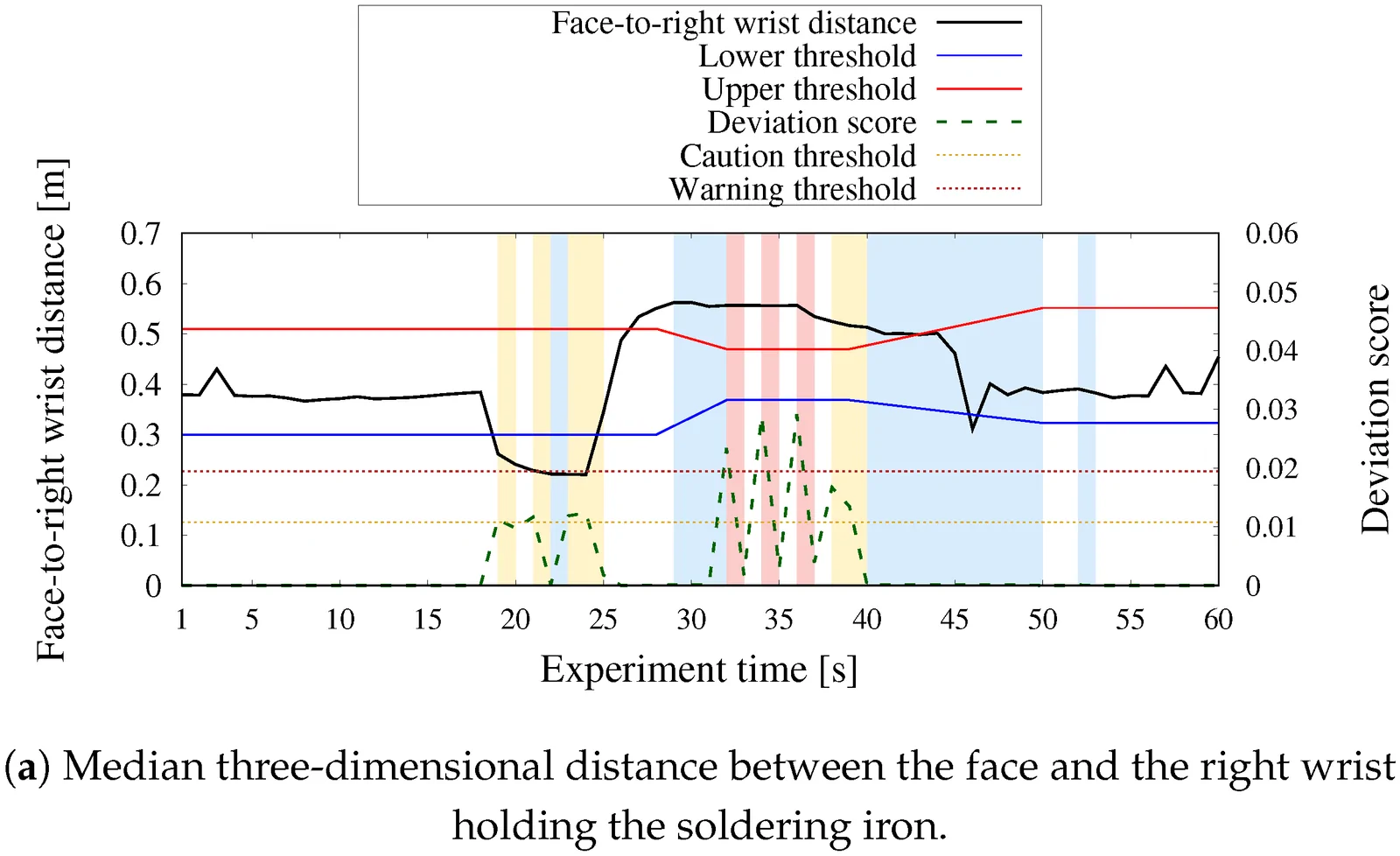

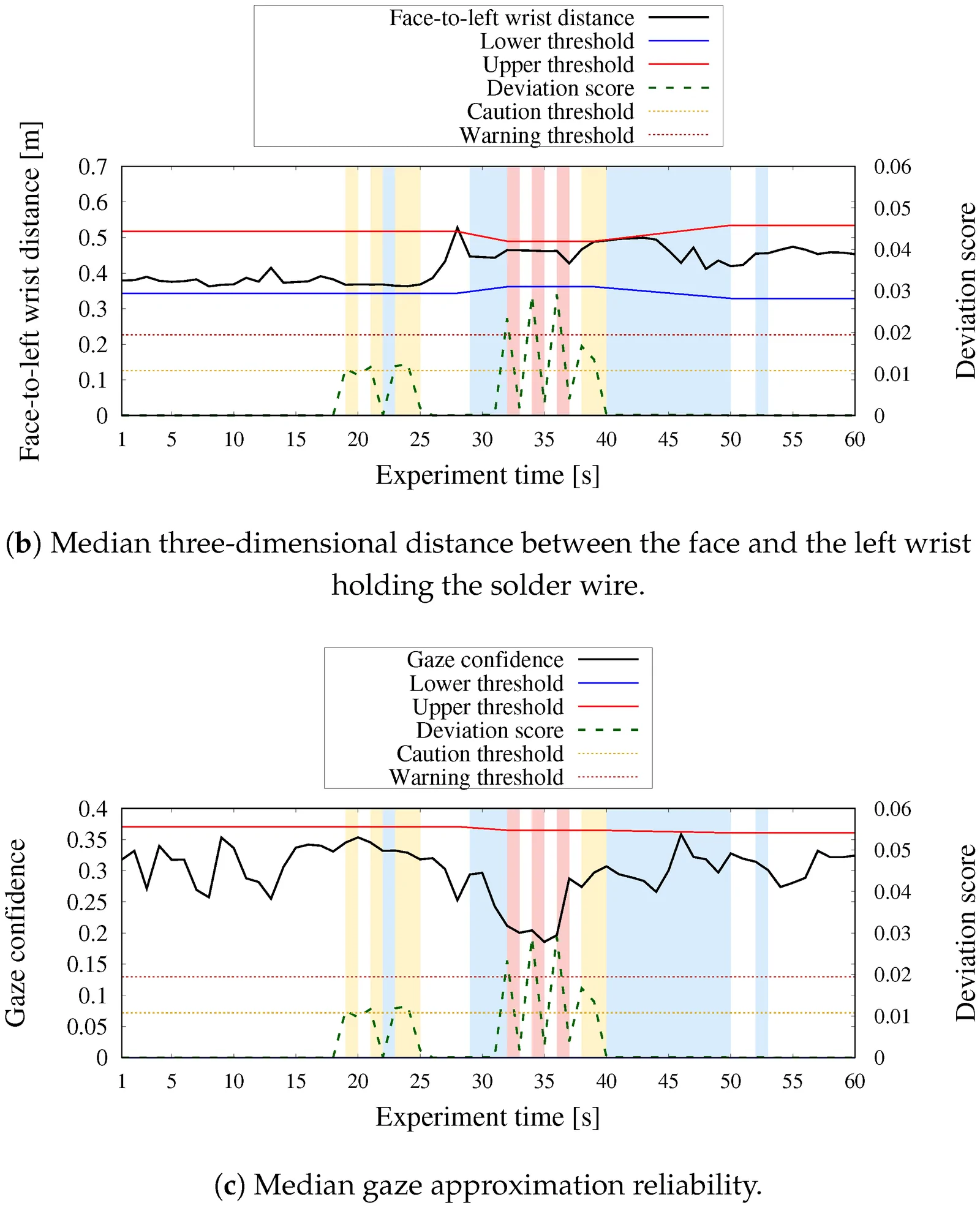
Temporal changes in face-to-wrist distance and gaze-related approximation features during a trial involving simulated unsafe-like movements.

**Figure 13:**
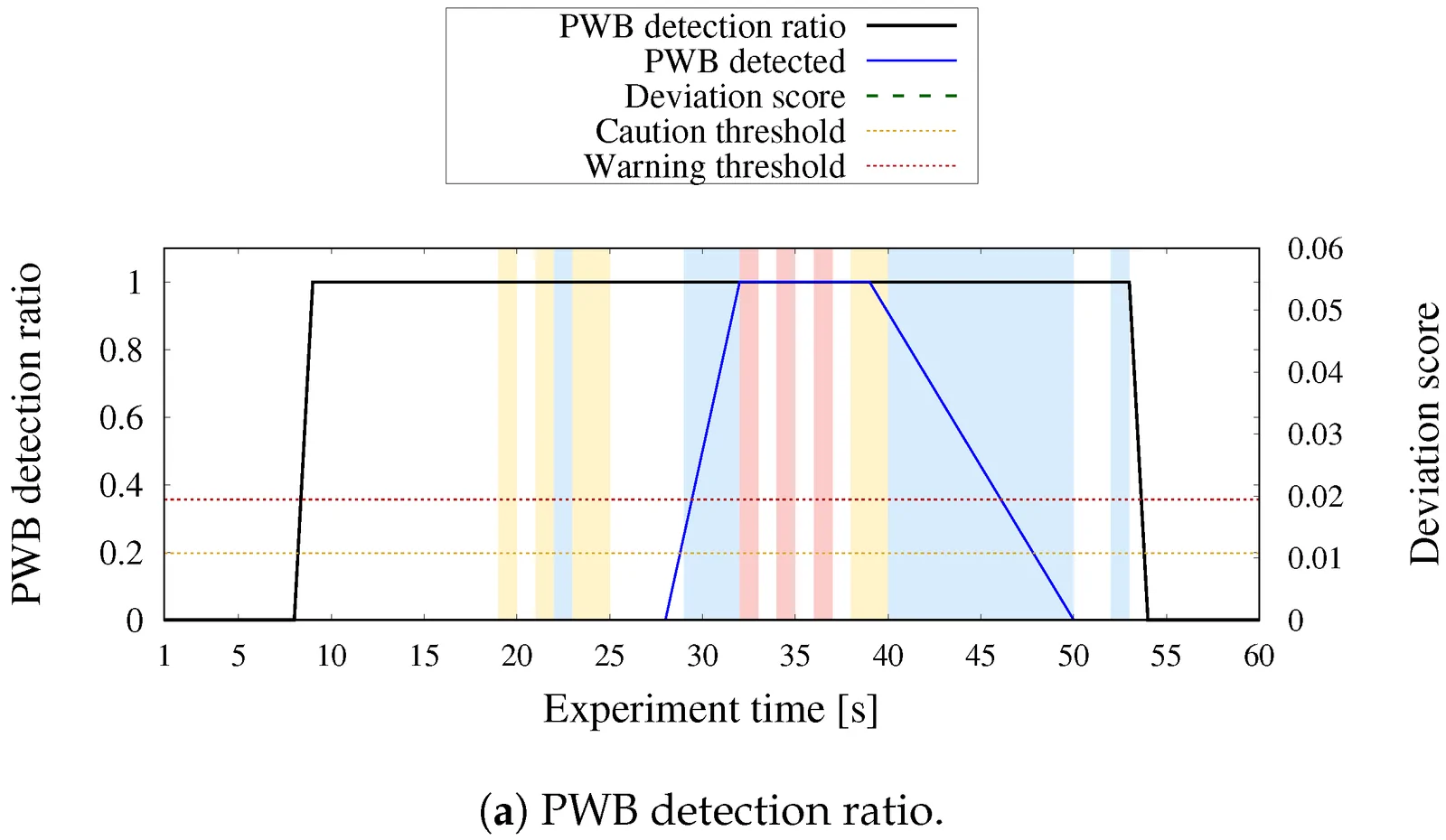

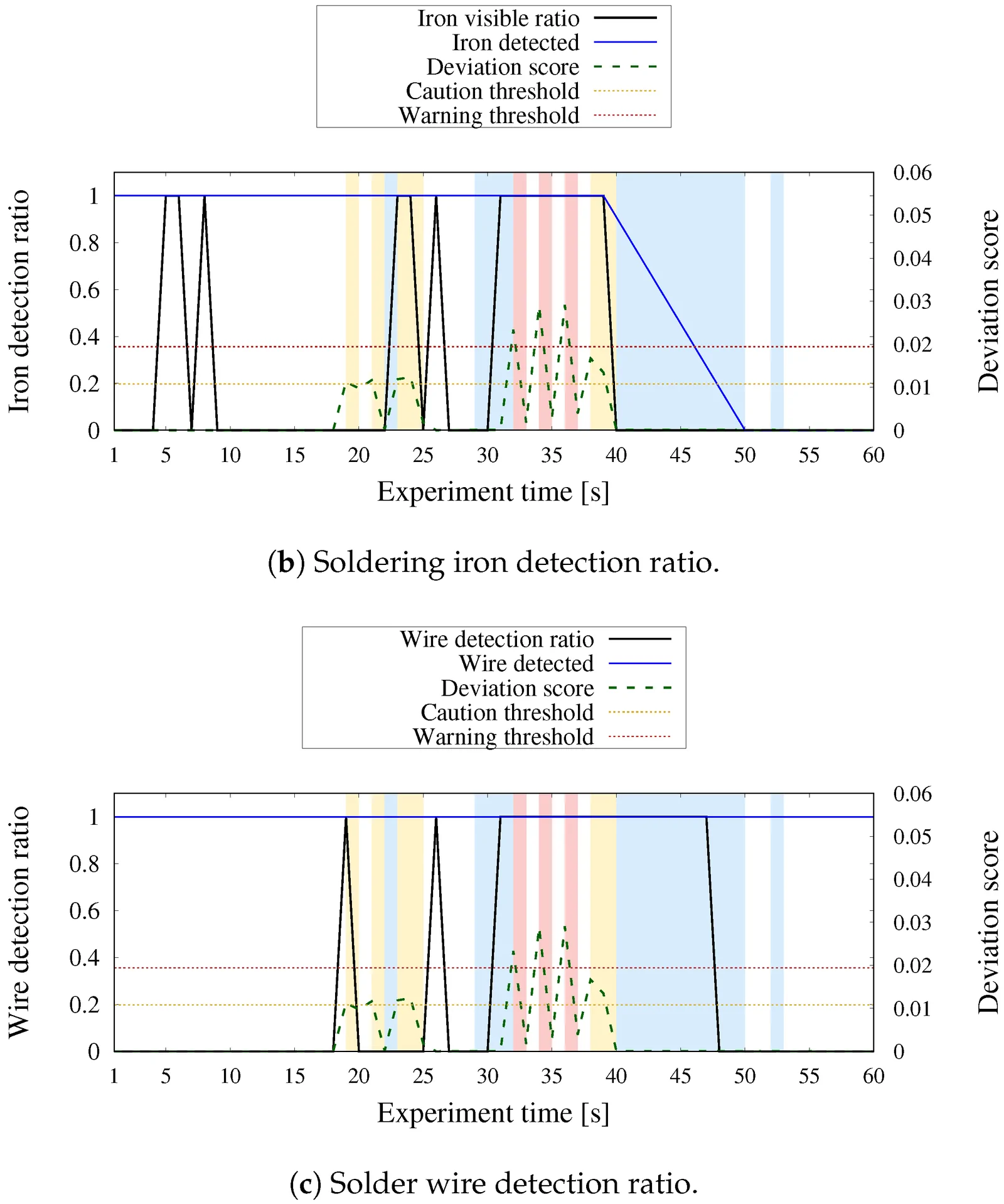
Temporal changes in object detection ratio during a trial involving simulated unsafe-like movements.

Figure 10 shows the estimated process phases and deviation scores for a trial involving simulated unsafe-like movements. In this experiment, actual accident events were not intentionally generated for ethical and safety reasons. Instead, movements resembling unsafe working states, such as unnatural upper-limb postures and excessive changes in the relationship between the face and the tool-holding hand, were simulated under controlled and safe conditions. In this trial, the phase-reliability assessment assigned 15 s of the entire 60 s interval to Uncertain Phase; these intervals were routed to review without automatic deviation-level classification. Among the remaining 45 s that were automatically assessed, 36 s were classified as Safe, 6 s as Caution, and 3 s as Warning. Among the automatically assessed intervals, the maximum deviation score was 0.0291. Intervals in the Caution score range (0.0108≤ deviation score <0.0194) were observed from 18 s to 23 s and from 37 s to 38 s. Intervals in the Warning score range (deviation score ≥0.0194) were observed at 31 s, 33 s, and 35 s. The estimated phase durations were 27 s for Preparation, 8 s for Active Soldering, and 10 s for Cleanup. The Uncertain Phase intervals occurred mainly from 28 s to 30 s and from 39 s to 48 s. Compared with the normal trial, the occurrence of Uncertain Phase segments suggests that the temporal consistency of the estimated process phases may have been reduced when simulated unsafe-like movements were inserted. This result suggests that the inserted movements affected not only individual feature values but also the consistency of the expected process structure. The repeated increases in the deviation score around the inserted movement intervals indicate that the proposed method responded to deviations from the normal feature patterns associated with each process phase. As shown in Figure 11, intervals were observed in which the elbow angles deviated from their phase-dependent reference ranges. The median elbow angle of the right arm holding the soldering iron ranged from $34.3^{\circ }$ to $143.4^{\circ }$. The right elbow angle exceeded the upper reference limit for 2 s, and the maximum deviation above the upper limit was approximately $9.1^{\circ }$. The median elbow angle of the left arm holding the solder wire ranged from $79.3^{\circ }$ to $173.7^{\circ }$. The left elbow angle exceeded the upper reference limit for 8 s, with a maximum deviation of approximately $40.4^{\circ }$ above the upper limit. These results indicate that the simulated unsafe-like movements were reflected as unnatural upper-limb postural changes, particularly in the left arm during the Active Soldering phase. As shown in Figure 12, changes in face-to-wrist distance and gaze-related approximation features were also observed. The three-dimensional distance between the face and the right wrist ranged from 0.220 m to 0.562 m. This feature deviated from the reference range for 15 s, including 5 s below the lower limit and 10 s above the upper limit. The maximum decrease below the lower limit was approximately 0.080 m, and the maximum increase above the upper limit was approximately 0.087 m. These deviations indicate that the positional relationship between the face and the tool-holding hand became different from that observed in normal trials. The three-dimensional distance between the face and the left wrist ranged from 0.363 m to 0.528 m. Only 1 s was detected outside the reference range, and the maximum deviation was approximately 0.011 m above the upper limit. In contrast, the gaze approximation reliability ranged from 0.186 to 0.358, and no interval was detected outside the reference range. Although gaze approximation reliability decreased locally around the high-score intervals, this feature did not directly exceed the reference limits in this trial. These results suggest that the increases in the deviation score were mainly associated with upper-limb posture and face-to-right-wrist distance rather than gaze approximation reliability. Temporal changes in the object detection ratio are shown in Figure 13. The detection ratios of the PWB, soldering iron, and solder wire varied between 0 and 1 according to the progress of the task and object visibility. The mean detection ratios were 0.750 for the PWB, 0.250 for the soldering iron, and 0.317 for the solder wire. No interval was detected outside the corresponding reference ranges for these object detection ratio features. Therefore, the increase in the deviation score in this trial was mainly attributed to deviations in elbow angle and face-to-wrist distance rather than to changes in the object detection ratio. Based on the above results, the trial involving simulated unsafe-like movements showed increases in the deviation score and several Uncertain Phase segments, while actual accident events were not generated. These findings indicate that the proposed method can respond to simulated unsafe-like deviations from normal soldering behavior under controlled and safe experimental conditions.

## 4. Discussion

### 4.1. Performance Interpretation and Relation to Previous Work

The central concept of this study is to interpret unsafe-like states not as anomalies in a single feature but as deviations from the expected coordination among posture, hand motion, tool position, face orientation, and work-object visibility in the current process phase. RF with offline Viterbi smoothing achieved an Accuracy and Macro-F1 of 0.729 and a Weighted-F1 of 0.736. The phase-wise F1-scores were 0.857 for Active Soldering, 0.674 for Preparation, and 0.656 for Cleanup. The lower performance for Preparation and Cleanup is consistent with their relatively similar low-motion and tool-placement behaviors, whereas the distinctive joining operation made Active Soldering easier to distinguish. Because the final deviation score integrates phase-specific scores according to the smoothed phase probabilities, phase ambiguity can change the weighting of the reference intervals and propagate to the downstream assessment.

For deviation detection, the proposed method achieved a Binary-F1 of 0.772, an FPR of 0.283, an FNR of 0.082, and a Warning recall of 0.942 over the 160 automatically assessed seconds. The most serious safety-related error is a false negative in which a simulated unsafe-like deviation is classified as normal. False positives are also important because they can contribute to alarm fatigue, whereas excessive routing to Uncertain Phase increases the human review burden. The phase-wise analysis showed the highest Binary-F1 during Active Soldering (0.931), but Cleanup had a Binary-F1 of 0.250 and an FPR of 0.419. Normal completion movements, such as placing tools down, withdrawing the hands, and returning to a resting posture, may have temporarily departed from the present phase-conditioned reference intervals and contributed to false-positive predictions during Cleanup. Accordingly, the current performance is not sufficient to establish deployment-ready industrial safety monitoring and should be interpreted as a controlled laboratory feasibility result.

Recent studies provide useful quantitative context for this interpretation. Wang and Yan reported an action-recognition accuracy of 99% and a progress-prediction time error below 6.9% for a human-centric assembly task using integrated skeleton and object information [22]. In contrast, validation of vision-based ergonomic assessment in real manufacturing environments has shown substantially greater variation across workstations and environmental conditions. Agostinelli et al. reported, for example, a risk-level accuracy of 65.22% for one investigated production line, whereas the corresponding score-level accuracies were 33.78% for REBA and 30.43% for RULA [14]. Bonakdar et al. reported a correlation of 0.95 for back-angle measurements between markerless vision and reference motion-capture/IMU measurements, with postural ergonomic assessments agreeing with the marker-based system for 87% of the participants [27]. These results indicate that quantitative performance depends strongly on the target task, sensing modality, evaluation variable, and experimental environment. Because the tasks, datasets, class definitions, validation units, and metrics differ, the present values should not be interpreted as a direct numerical performance ranking against these studies.

The representative trials also illustrate the role of multiple features. In the normal trial, the experimental-state label remained Safe, the deviation score remained at 0.0, and no Uncertain Phase segment was observed. In the representative trial containing simulated unsafe-like movements, the principal contributions were deviations in the upper-limb posture and the face-to-tool-holding-hand relationship rather than object-detection ratios or gaze-approximation reliability. This result supports feature-level interpretation but does not establish that the same contributing features will generalize to other workers or incident types.

RF was selected for the engineered tabular multimodal features and limited number of sessions in the present dataset as it provides nonlinear modeling, class probabilities for soft phase integration, and feature-level interpretation. This choice does not imply that RF is generally superior to deep temporal models. Future comparisons should include LSTM, temporal convolutional networks, and Transformer-based approaches using larger, participant-diverse datasets and participant-wise validation.

### 4.2. Biomimetic Interpretation and Sensing Choices

The biomimetic contribution of this study is functional rather than morphological and lies in translating the context-dependent perceptual-motor coordination observed in skilled manual work into an engineering information-processing structure. In precision soldering, workers coordinate upper-body posture, hand motion, tool position, face orientation, and tool-workpiece relationships according to the current task phase. The proposed framework represents this organization as phase-dependent expected coordination patterns and evaluates multimodal deviations from these patterns. Thus, the framework does not reproduce biological morphology or neural mechanisms; rather, it functionally abstracts the context-dependent coordination between perception and movement observed in skilled work.

The same joint configuration or tool trajectory can have different meanings in different task phases. Accordingly, the phase variable can be regarded as a compact representation of task context, while the body-tool-workpiece features describe the coordination expected in that context. A similar phase-conditioned reference structure may be useful as a monitoring or shared-control layer in an anthropomorphic or robotic system, where context-appropriate movement must be distinguished from deviation. However, robotic control was neither implemented nor evaluated in this study.

Compared with a simpler two-dimensional vision configuration, RGB-D sensing provides depth information that enables three-dimensional spatial relationships among the operator, tools, and workpiece to be represented, although it requires additional depth processing and calibration and remains sensitive to visual occlusion [37,38]. Wearable inertial sensors can capture body-attached kinematics without requiring a continuous visual line of sight, but they require attachment to the operator and appropriate sensor-to-segment calibration [39,40]. Recent direct comparisons between markerless vision and inertial sensing further indicate that the modalities offer different advantages depending on the target measurement and application, rather than demonstrating universal superiority of either modality [27]. The RGB-D configuration was selected because it enables body-tool-workpiece relationships to be observed without attaching sensing devices to the operator. Its use therefore represents a trade-off between access to three-dimensional spatial information and the additional requirements associated with depth processing, calibration, and visual occlusion. The functional biomimetic mapping from skilled manual behavior to the proposed engineering framework is summarized in Table 9.

### 4.3. Application Perspective

The proposed framework can trace an assessment to the process phase, body part, and feature category, which may support retrospective safety review and educational feedback. At 30 fps, the 30-frame analysis window contains approximately 1.0 s of observations, and the 10-frame step provides an update interval of approximately 0.33 s. However, this interval represents the update interval of the analysis windows and not the processing time of the complete system, which included image acquisition, pose estimation, object recognition, three-dimensional coordinate calculation, phase estimation, and deviation evaluation. The present implementation was applied to previously recorded data and used Viterbi smoothing as offline temporal post-processing.

One-second median aggregation suppresses frame-level fluctuations and outliers but can attenuate very brief sub-second events. Therefore, the current system was not designed or validated as an immediate emergency-warning system for sudden contact with a hot soldering iron tip. Practical use for immediate safety intervention will require a separate low-latency processing path, measurement of the end-to-end processing time on the intended deployment hardware, and validation in representative workplace environments.

### 4.4. Limitations

This study has several limitations. First, although participant-wise leave-one-participant-out evaluation was used for phase estimation, the dataset included only five participants and ten 60 s laboratory sessions. The small sample does not establish population-level generalization. Moreover, for each participant under evaluation, the phase-conditioned 5th–95th percentile reference intervals were constructed from the normal trials of the other four participants and may include both within-phase variation and between-participant differences in body size, soldering experience, work speed, and working style. Because the independent effect of skill level could not be statistically separated, these intervals should be interpreted as descriptive references for the present dataset rather than as population-level safety limits.

Second, the simulated unsafe-like movements were scripted and deliberately performed under controlled and safe conditions. They do not reproduce unconscious errors, inattention, fatigue, startle responses, accidental contact, or the diversity and severity of actual workplace incidents. In addition, the number of development trials used to determine the deviation-detection operating point was limited, and the stability of the selected thresholds should be examined using larger independent datasets. Further validation in actual workplaces under the supervision of occupational-safety experts is required. The annotation-reliability analysis used two members of the same laboratory, not multiple independent occupational-safety experts; therefore, the reported agreement statistics support labeling consistency within this study but do not constitute external expert validation of the experimental-state labels.

Third, the tool-holding protocol, camera position, workstation, and lighting conditions were fixed. Although the known-distance test confirmed stable depth measurements within the investigated camera-to-workspace range, robustness to controlled changes in keypoint occlusion, soldering smoke, illumination, reflective conditions, and camera position was not systematically evaluated. Joint-loss rates, object-visibility ratios, and detection-confidence features can represent missing or unstable observations and can contribute to review routing. The current Uncertain Phase mechanism can therefore conservatively route low-confidence observations to review but should not be interpreted as evidence of environmental robustness.

Although the YOLO dataset was separated by participant across training, validation, and testing, the test set contained images from only one participant and therefore does not establish broad cross-participant object-segmentation generalization. In addition, the solder wire class showed substantially lower recall, mAP, and semantic IoU than the PWB and soldering iron classes. Consequently, spatial features that depend on reliable solder-wire localization should be interpreted cautiously, and the present results do not establish robust solder-wire tracking under different participants or workplace conditions. A downstream ablation excluding solder-wire-dependent features was not performed, so the independent contribution of this comparatively weak component to the final deviation-detection result remains unresolved.

The final deviation-detection metrics were aggregated over three participant-level held-out trials. Because the number of independent held-out participants is small, the aggregate 180 s time series should not be interpreted as 180 independent experimental units, and participant-level variability in Binary-F1, FPR, FNR, and Warning recall is not established by the present study. In addition, the matched-subset comparison excludes the intervals selected by the proposed review gate for all methods. This provides identical samples for the reported comparison but makes the subset selection endogenous to the proposed framework. A native 100%-coverage comparison and a systematic risk-coverage analysis were not performed and are required to quantify the trade-off between automatic coverage, missed detections, and false alarms independently of the selected operating point.

Finally, one-second median aggregation and offline Viterbi smoothing prioritize temporally stable analysis and can attenuate very brief sub-second events. End-to-end processing time was not benchmarked; therefore, the present implementation was not validated as a real-time emergency-warning system. Direct synchronized comparisons with two-dimensional-only vision or wearable-sensor configurations were also not performed.

## 5. Conclusions

This study proposed a phase-conditioned deviation-detection framework for seated manual soldering using an RGB-D camera. The framework estimated Preparation, Active Soldering, and Cleanup from three-dimensional body posture, hand movement, task-related object relationships, object visibility, detection confidence, and face-orientation-related observations. Intervals without sufficient phase confidence were assigned to Uncertain Phase and routed to review rather than being given an automatic experimental-state label.

RF with Viterbi smoothing achieved an Accuracy and Macro-F1 of 0.729 for phase estimation. For deviation detection, the proposed method achieved a Binary-F1 of 0.772, an FNR of 0.082, and a Warning recall of 0.942 over the automatically assessed intervals, while 11.1% of the evaluation intervals were routed to review. The phase-wise and feature-level results showed that the framework represented unsafe-like observations as phase-dependent multivariate deviations rather than as exceedances of a single global threshold.

Taken together, these findings demonstrate the feasibility of a retrospective, phase-conditioned analysis of scripted simulated unsafe-like movements under controlled laboratory conditions. They do not constitute validation of actual danger, injury risk, real-time emergency response, or industrial deployment performance. Future work should use larger and more diverse participant samples, independent threshold-development data, naturalistic expert-reviewed workplace scenarios, and end-to-end runtime evaluation.

## Figures and Tables

**Table 1:** Depth-measurement accuracy of the Intel RealSense D455 under the experimental configuration. MAE, mean absolute error; RMSE, root mean square error.

Reference [m]	Measured [m]	Bias [mm]	MAE [mm]	RMSE [mm]
0.50	0.4970	−3.00	3.00	3.00
0.60	0.5998	−0.19	0.25	0.40
0.70	0.6937	−6.32	6.32	6.34

**Table 2:** Definitions of soldering process phases.

Phase	Definition
Preparation	The phase preceding Active Soldering, including tool preparation and other actions required to initiate the work.
Active Soldering	The primary operational phase, including approaching the PWB, heating, supplying solder, and inspecting the joints.
Cleanup	The phase following Active Soldering, including tool tidying, workpiece organization, and other actions associated with task completion.
Uncertain Phase	A phase-unassignable state in which a time window cannot be assigned to Preparation, Active Soldering, or Cleanup with sufficient confidence. This state is not treated as a deviation label or a ground-truth process-anomaly label; instead, it is used as a reliability flag for phase estimation and is output as a review-required interval.

**Table 3:** Categories and representative features used for deviation detection.

Category	Representative Features
Right Elbow	Median angle of the right elbow
Left Elbow	Median angle of the left elbow
Right Wrist	Median velocity of the right wrist, median distance between the face and the right wrist, and median distance between the right wrist and the soldering iron
Left Wrist	Median velocity of the left wrist, median distance between the face and the left wrist, and median distance between the left wrist and the solder wire
Face Safety	Median distance between the face and the soldering iron
Tool Operability	Median distance between the soldering iron and the PWB, median distance between the solder wire and the PWB, and median distance between the soldering iron tip and the solder wire tip
Workspace Visibility	Visibility ratios of the PWB, soldering iron, and solder wire and median detection confidence for each object
Gaze-Related Approximation Features	Gaze-estimation success rate, median gaze-approximation reliability, and median head yaw, pitch, and roll

**Table 4:** Class-wise segmentation-mask and overall object-detection and instance-segmentation performance of the YOLO model on the participant-disjoint test set.

Evaluation Scope	Metric Type	Precision	Recall	mAP@0.5	mAP@0.5:0.95	Semantic IoU
PWB	Segmentation mask	0.998	1.000	0.995	0.956	0.881
Soldering iron	Segmentation mask	0.958	1.000	0.995	0.794	0.825
Solder wire	Segmentation mask	0.474	0.200	0.205	0.023	0.356
Overall	Bounding box	0.863	0.828	0.866	0.704	N/A
Overall	Segmentation mask	0.810	0.733	0.732	0.591	0.687

**Table 5:** Comparison results of phase-estimation methods.

Method	Accuracy	Macro-F1	Weighted-F1	Preparation F1	Active Soldering F1	Cleanup F1
RF with Viterbi smoothing	0.729	0.729	0.736	0.674	0.857	0.656
RF	0.643	0.643	0.653	0.535	0.850	0.542
SVM-RBF	0.608	0.608	0.613	0.503	0.734	0.587
SVM-linear	0.598	0.593	0.600	0.532	0.718	0.531

**Table 6:** Phase-wise performance of RF with Viterbi smoothing. FPR, false-positive rate.

Phase	Accuracy	Precision	Recall	F1-Score	FPR
Preparation	0.774	0.644	0.707	0.674	0.194
Active Soldering	0.905	0.962	0.772	0.857	0.018
Cleanup	0.778	0.618	0.699	0.656	0.187

**Table 7:** Matched-subset comparison results of the proposed deviation-detection method with baseline methods. FPR, false-positive rate; FNR, false-negative rate.

Method	Native Auto. [s]	Common Excl. [s]	Matched Eval. [s]	Accuracy	Macro-F1	Binary-F1	FPR	FNR	Warning Recall
Proposed	160	20	160	0.750	0.511	0.772	0.283	0.082	0.942
Baseline A global	180	20	160	0.661	0.477	0.565	0.216	0.453	0.482
Baseline B heuristic	180	20	160	0.578	0.432	0.635	0.448	0.156	0.696

**Table 8:** Phase-wise binary performance of the proposed deviation-detection method.

Ground-Truth Phase	Input [s]	Eval. [s]	Review	Review Rate	Binary Accuracy	Precision	Recall	Binary-F1	FPR	FNR
Preparation	60	57	3	0.050	0.860	0.429	1.000	0.600	0.157	0.000
Active Soldering	63	57	6	0.095	0.877	0.959	0.904	0.931	0.400	0.096
Cleanup	57	46	11	0.193	0.609	0.143	1.000	0.250	0.419	0.000

**Table 9:** Functional biomimetic mapping from skilled manual behavior to the engineering framework.

Biological/Behavioral Principle	Functional Abstraction	Engineering Realization in This Study
Skilled manual action depends on task context and phase	The same posture or tool trajectory can have different meanings according to the current process state	Three-phase process estimator followed by phase-conditioned deviation interpretation
Human skilled action coordinates posture, hands, tools, and visual orientation	Task state is represented by a multimodal coordination pattern rather than a single feature	Integration of body, hand, object, visibility, and face-orientation-related features
Ambiguous perception motivates additional checking rather than immediate commitment	Low-confidence context should be deferred instead of forcing a state assignment	Uncertain Phase reliability flag and routing of the interval to human review
Expected coordination changes as the work proceeds	Normality is context dependent rather than global	Phase-conditioned reference intervals and probability-weighted integration of phase-specific deviation scores

## Data Availability

The data used in this study are not publicly available due to participant privacy considerations, as the raw RGB-D recordings contain potentially identifiable participant images and laboratory-workspace information.
